# A review of the evidence base for utilizing Child-Pugh criteria for guiding dosing of anticancer drugs in patients with cancer and liver impairment

**DOI:** 10.1016/j.esmoop.2021.100162

**Published:** 2021-06-05

**Authors:** C. Palmieri, I.R. Macpherson

**Affiliations:** 1 Department of Molecular and Clinical Cancer Medicine, Institute of Systems, Molecular and Integrative Biology, University of Liverpool, Liverpool, UK; 2 Academic Department of Medical Oncology, The Clatterbridge Cancer Centre NHS Foundation Trust, Liverpool, UK; 3 Institute of Cancer Sciences, University of Glasgow, Glasgow, UK

**Keywords:** liver impairment, Child-Pugh criteria, dosing, anticancer drugs

## Abstract

As the liver is vital for the metabolism of many anticancer drugs, determining the correct starting doses in cancer patients with liver impairment is key to safe prescription and prevention of unnecessary adverse effects. Clinicians typically use liver function tests when evaluating patients; however, prescribing information and summaries of product characteristics often suggest dosing of anticancer drugs in patients with liver impairment based on the Child-Pugh criteria, even though the criteria were not developed for this purpose. In this review, we assessed all the oncological small molecule and cytotoxic drugs approved by the United States Food and Drug Administration (FDA) over a 5-year period from 2014 to 2018. The various entry criteria related to these drugs—with respect to hepatic function—in key pivotal studies were compared with their approved dosing recommendations found in prescribing information and summaries of product characteristics. We found that 46% of drugs have dosing recommendations based on Child-Pugh criteria alone, despite the fact that only 8% of these drugs were tested within studies that used the Child-Pugh criteria as entry criteria. Moreover, we note that the data used to make recommendations based on Child-Pugh criteria are typically from small studies that may lack an appropriate patient population. We propose that these findings, along with details surrounding the development of the Child-Pugh criteria, call into question the validity and appropriateness of using Child-Pugh criteria for dosing recommendations of anticancer drugs.

## Highlights

• Dosing information for anticancer drugs in patients with liver impairment is often based on the Child-Pugh criteria.
• Clinical trials and clinicians typically use liver function tests when evaluating patients.
• Of the 39 oncologic drugs examined, almost half (46%) had dosing recommendations based on Child-Pugh criteria alone.
• We question whether using Child-Pugh criteria for dosing recommendations of anticancer drugs is the best approach.

## Introduction

The liver is critically important for the metabolism and excretion of many anticancer drugs^1^^,^^2^ and liver disease and/or liver impairment in patients with cancer can alter drug pharmacokinetics, necessitating dose modification. Depending on the drug administered, impairment may slow the breakdown of active or toxic metabolites (i.e. increasing drug toxicity) or the activation of prodrugs (i.e. decreasing drug effectiveness).^3^ Additionally, varying etiologies of liver impairment, such as liver metastases, previous anticancer treatments, cirrhosis, or hepatitis, may differently impact the pharmacokinetics of drugs.^3^^,^^4^ The narrow therapeutic index of many anticancer drugs,^5^ the possible toxicities from incorrect dosing, and the potential loss of efficacy from underdosing, all demonstrate the importance of determining the correct starting dose of drugs in patients with cancer and liver impairment.

The European Medicines Agency (EMA) and United States Food and Drug Administration (FDA) have both recognized the lack of optimal biomarkers that can be used to define categories of liver impairment with respect to effects on drug pharmacokinetics.^6^^,^^7^ Despite extensive efforts, no single measure or group of measures has gained widespread clinical use for the estimation of how hepatic impairment will affect drug pharmacokinetics and/or pharmacodynamics in a given patient.^6^^,^^7^ As such, the EMA and FDA have allowed the use of the Child-Pugh criteria (Supplementary Table S1, available at https://doi.org/10.1016/j.esmoop.2021.100162)^6^^,^^7^ to categorize the degree of patients’ hepatic impairment. An important caveat in the EMA and FDA guidelines is that liver impairment must be the cause of alterations in Child-Pugh components (i.e. clinicians should rule out other underlying disease processes as the cause of Child-Pugh alterations).^6^^,^^7^ However, the causality of changes in parameters contributing to Child-Pugh score can be difficult to discern in the context of cancer. The EMA guidance also makes it clear that Child-Pugh criteria should only be used for classification purposes if a study includes patients with an adequate range of serum albumin concentrations (decreased from normal), as well as bilirubin concentrations and prothrombin times (increased from normal).^6^

The Child-Pugh criteria are composed of clinical and biochemical features (including encephalopathy, ascites, serum albumin level, serum bilirubin level, and international normalized ratio; Supplementary Table S1, available at https://doi.org/10.1016/j.esmoop.2021.100162).^8^ These criteria were originally developed to assess operative risk in patients undergoing surgical portosystemic shunt due to chronic liver disease and are now routinely used as a prognostic measure in patients with chronic liver disease and cirrhosis.^8^ As such, these criteria were not designed or validated for finessing the dosing of systemic cancer therapies in patients with liver dysfunction secondary to metastatic cancer. Despite the known weaknesses associated with Child-Pugh criteria, the FDA and EMA guidance for dosing in patients with liver impairment is often characterized using Child-Pugh criteria. Notably, others have criticized the clinical applicability of information provided per FDA or EMA guidance for patients with cancer and hepatic impairment within the prescribing information (in the United States) and summaries of product characteristics (SmPCs; in Europe).^9^^,^^10^

In this narrative review, we aimed to identify which criteria (Child-Pugh or other) were used within the prescribing information or SmPCs to guide dosing recommendations of 39 oncologic drugs, and whether these criteria were supported by peer-reviewed pharmacological studies. In doing so, we explored the differences between criteria used for approved dosing recommendations in prescribing documents with hepatic function inclusion criteria within the pivotal clinical trials (i.e. those trials referred to within those same prescribing information documents or SmPCs).

## Methods

This review was designed to assess all oncologic small molecule and cytotoxic drugs approved by the FDA over a selected 5-year period from 2014 to 2018. This list of treatments was derived from the FDA website. For each drug, guidance regarding prescribing and dosing in the presence of liver impairment was collated from prescribing information (FDA) and SmPCs (EMA; where available). Pivotal studies referred to within the prescribing information and SmPCs were reviewed to identify inclusion and exclusion criteria related to liver function used for each drug; if available (i.e. published with the manuscript), the protocols of clinical studies were reviewed to ensure that complete and detailed criteria were obtained (Supplementary Table S2, available at https://doi.org/10.1016/j.esmoop.2021.100162). Pharmacokinetic studies in the prescribing information and SmPCs were included in this narrative review if Child-Pugh criteria were used in the studies. Additionally, searches were made on PubMed for articles published before 30 March 2019. These searches included the name of each drug AND ‘Child Pugh’; additional searches used the name of each drug AND ‘liver function.’ Papers assessing the effects of liver impairment on drug dosing were reviewed if they met the following criteria: (i) Child-Pugh criteria were used as the inclusion criteria and (ii) studies were published in English. Any relevant information or studies (published in English) regarding the use of Child-Pugh criteria referred to within the prescribing information or SmPCs were also reviewed. A separate search on clinicaltrials.gov was completed to identify pharmacokinetic studies that may not have been included in the PubMed search. Studies from this search are included in Supplementary Table S3, available at https://doi.org/10.1016/j.esmoop.2021.100162, irrespective of the liver impairment criteria used; studies were recorded irrespective of the results of the PubMed search. The search terms used to search clinicaltrials.gov were the drug name AND ‘Child-Pugh’ OR ‘liver impairment’ OR ‘hepatic impairment’. If the published studies were not directly identified on clinicaltrials.gov, then the NCT numbers for these studies were used to locate any corresponding publications. Data searches and extraction were carried out by IRM and CP.

### Patient and public involvement statement

Due to the procedures needed to conduct this review, it was not considered a suitable option to include patient/public involvement.

## Results

### Overview of oncologic small molecule and cytotoxic drugs approved by the FDA

**Figure 1:**
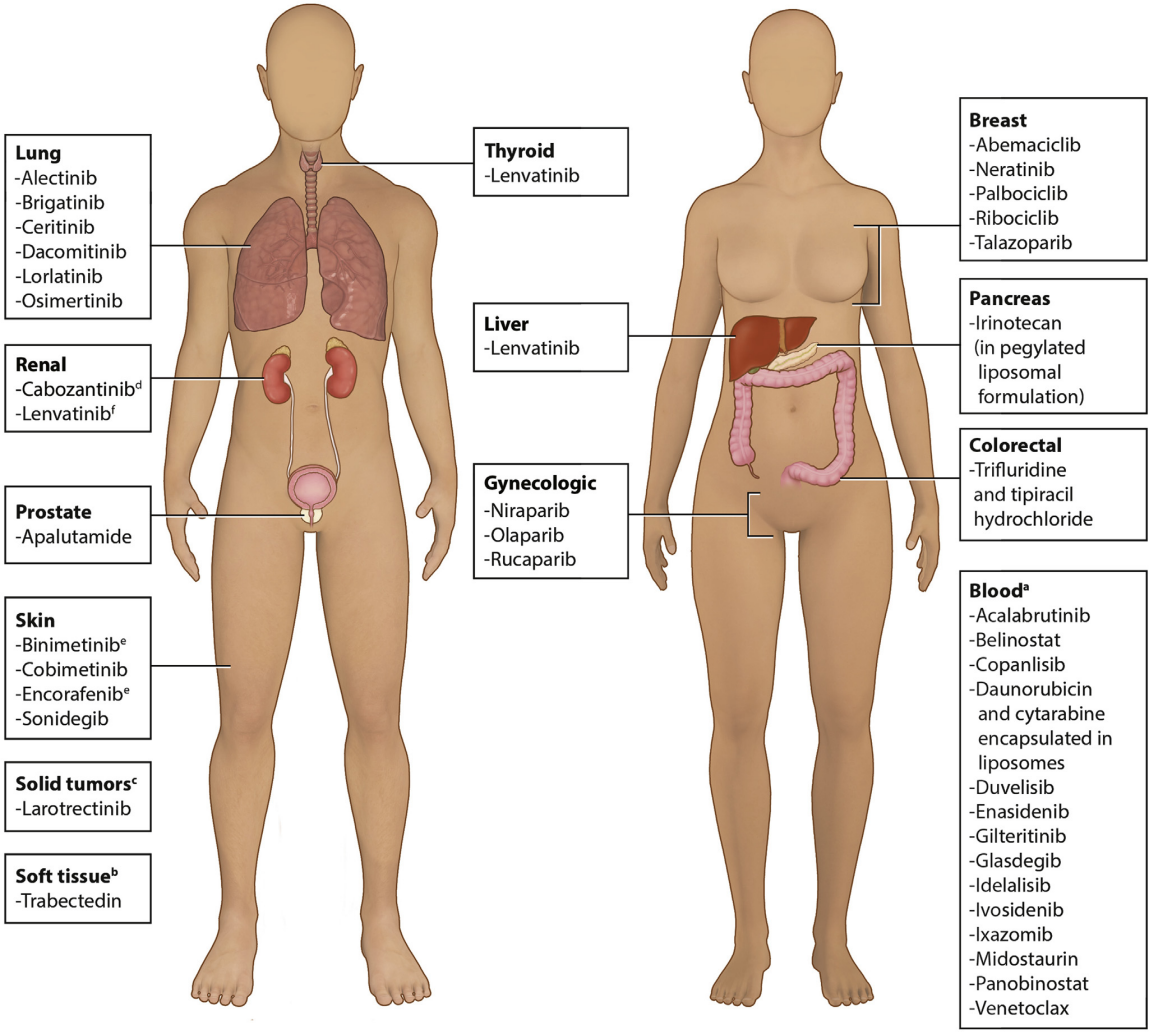
FDA-approved (2014-2018) oncologic small molecule and cytotoxic drugs by broad indication. Some therapies are indicated for >1 cancer. Some therapies are used in combination with other therapies (combination therapy is not listed in the figure). FDA, United States Food and Drug Administration. ^a^ Includes lymphoma, leukemia, and myeloma. ^b^ Includes liposarcoma, and leiomyosarcoma. ^c^ Includes solid tumors that: have neurotrophic receptor tyrosine kinase gene fusion without a known acquired resistance mutation; are metastatic or where surgical resection is likely to result in severe morbidity; have no satisfactory alternative treatments or that have progressed following treatment. ^d^ Two formulations are available: Cabometyx® and Cometriq®; Cabometyx obtained approval for renal cell carcinoma between 2014 and 2018, approval for hepatocellular carcinoma occurred in 2019; FDA approval of Cometriq occurred before 2014. ^e^ Encorafenib and binimetinib are indicated in combination. ^f^ Lenvatinib in combination with everolimus.

From 2014 to 2018, 39 oncologic small molecule and cytotoxic drugs were approved by the FDA (Supplementary Table S2, available at https://doi.org/10.1016/j.esmoop.2021.100162). Of these drugs, lenvatinib was approved for three indications (locally recurrent or metastatic, progressive, radioactive iodine-refractory differentiated thyroid cancer; first-line treatment of patients with unresectable hepatocellular carcinoma (HCC); and in combination with everolimus, for the treatment of patients with advanced renal cell carcinoma following one prior antiangiogenic therapy. All other drugs were approved for a single indication during the defined time period. Indications spanned 13 broad categories (Figure 1); 14 drugs were approved to treat blood cancers, six were approved to treat lung cancers, and five were approved for use in breast cancer (Figure 1). No more than four drugs were approved for each remaining category of cancer (i.e. gynecologic, liver, kidney, soft tissue, pancreas, skin, colorectal, thyroid, prostate, and solid tumors) (Figure 1).

### Dosing guidance for patients with liver impairment: prescribing information and SmPCs

**Figure 2:**
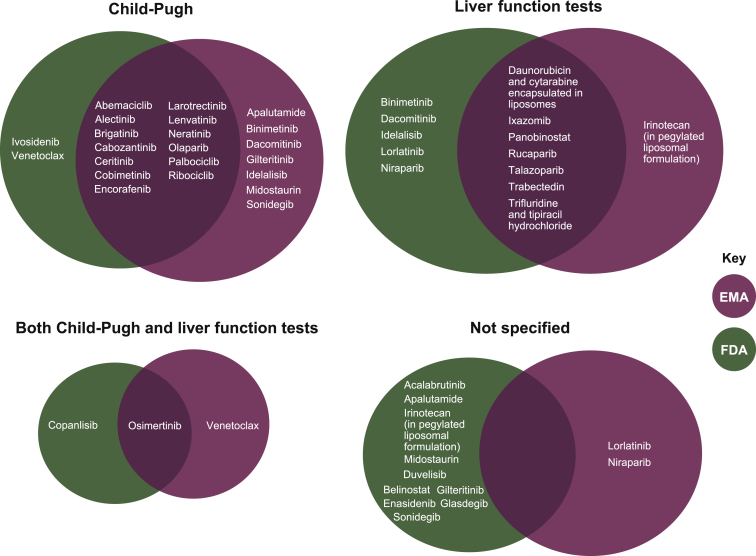
Comparison of criteria used for dosing recommendations made by the EMA and FDA.^a^

According to the prescribing information and SmPCs of the 39 oncologic small molecule and cytotoxic drugs examined, recommendations for initial dosing in patients with liver impairment were based on liver function tests alone for 26% (10/39) of drugs and Child-Pugh scores alone for 46% (18/39) of drugs (Figure 2; Supplementary Table S2, available at https://doi.org/10.1016/j.esmoop.2021.100162). Guidance was not specified (i.e. criteria defining liver impairment were not specified or specific guidance was not given) for 13% (5/39) of drugs; and guidance was based on both Child-Pugh scores and liver function tests for the remaining drugs (15%; 6/39) (Figure 2; Supplementary Table S2, available at https://doi.org/10.1016/j.esmoop.2021.100162).

**Table:** 

Criteria (%)	FDA (M^b^ = 39)	EMA (M^b^ = 32)	FDA and EMA (M^b^ = 39)
Child-Pugh (only)	15 (38)	20 (63)	18 (46)
Liver function tests (only)	12 (31)	8 (25)	10 (26)
Both Child-Pugh and liver function tests	2 (5)	2 (6)	6 (15)^c^
Not specified	10 (26)	2 (6)	5 (13)

EMA, European Medicines Agency; FDA, United States Food and Drug Administration; SmPC, summary of product characteristics.

^a^ The following drugs did not have a publicly available SmPC (through August 2020) and therefore do not appear under the EMA or ‘both’ portions of the graph: acalabrutinib, belinostat, copanlisib, duvelisib, enasidenib, glasdegib, and ivosidenib.

b M equals the number of approved drugs by the FDA or EMA, respectively; percentage based on the total number of drugs.

c This number refers to drugs that had dosing guidance based on both Child-Pugh and liver function test criteria; please note, this includes drugs that had dosing guidance based on Child-Pugh criteria in the prescribing information (FDA) and liver function tests in the SmPCs (EMA) or vice versa.

Recommendations from the FDA and EMA are summarized separately because 7 of the 39 treatments did not have publicly available SmPCs at the time of manuscript preparation (August 2020). Based on the various drugs’ prescribing information (FDA) documents, 31% (12/39) of drugs had recommendations based on liver function tests alone, 38% (15/39) had recommendations based on Child-Pugh scores alone, and the criteria for recommendations (or recommendations themselves) were not specified for 26% (10/39) of drugs (Figure 2). Only copanlisib and osimertinib had recommendations based on both Child-Pugh scores and liver function tests (Figure 2). Of the 32 with available SmPCs, these documents included dosing recommendations based on Child-Pugh scores alone in 63% (20/32) of drugs and liver function tests alone in 25% (8/32) of drugs. Specific recommendations/criteria for recommendations were not explicit in 6% (2/32) of drugs, and only osimertinib and venetoclax had recommendations for both Child-Pugh scores and liver function tests.

### Comparison of pivotal trial data and dosing guidance summarized in prescribing information and/or SmPCs

Of the 75 pivotal published studies referred to within the prescribing information and SmPCs of reviewed drugs, the full protocol was published along with the manuscript 71% (53/75) of the time (Supplementary Table S2, available at https://doi.org/10.1016/j.esmoop.2021.100162). Protocols, manuscripts (where the protocols were not available), or other sources (if the study was not published), were reviewed to identify entry criteria related to hepatic function. Review of this information revealed that study protocols referenced using Child-Pugh criteria for only 8% (3/39) of drugs [i.e. in pivotal studies of lenvatinib, cabozantinib, and ribociclib (exclusion criteria only)]. In the case of lenvatinib, the Child-Pugh criteria were used in an HCC trial protocol (REFLECT); however, liver function tests were also used as inclusion criteria.^11^ For cabozantinib, the METEOR study in renal cell carcinoma excluded patients who were classified as having Child-Pugh class B or C; however, inclusion criteria were based on liver function tests.^12^ And although the CELESTIAL study in HCC (cabozantinib was indicated for HCC in 2019) allowed the inclusion of patients classified as having Child-Pugh class A, liver function tests were also included as part of the entry criteria.^13^ Overall, for 92% (22/24) of drugs where Child-Pugh scores were used for specific dosing recommendations in either the prescribing information or SmPCs (or both), Child-Pugh criteria had not been used in the pivotal trial.

### Liver impairment—as assessed by Child-Pugh criteria—and drug pharmacokinetics

**Figure 3:**
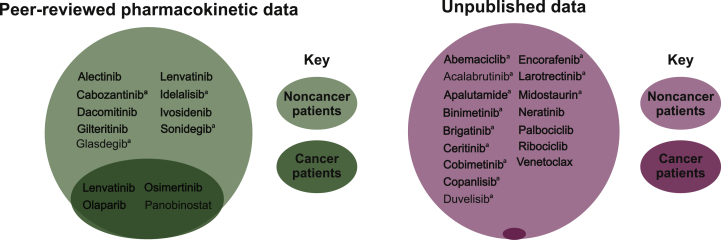
Summary of all pharmacokinetic studies in patients with liver impairment assessed by Child-Pugh criteria. Drug names in bold represent those drugs for which dosing guidelines in the prescribing information and/or the SmPCs use Child-Pugh criteria. ^a^ Patients were assumed to be ‘noncancer patients’ because no details on underlying hepatic impairment or the specific patient populations were presented in the pharmacokinetic studies.

The 24 drugs for which approved dosing recommendations were based on Child-Pugh criteria (i.e. in prescribing information and/or SmPCs; Figure 3; those set in bold font) all had pharmacokinetic studies in patients where liver impairment was characterized using the Child-Pugh criteria. However, studies for 14 of these drugs were not published, and information on the types of patients [i.e. patients with cancer (and what type of cancer) or without cancer] included in these 14 studies were limited (Figure 3). Of the remaining 10 drugs with a peer-reviewed publication describing dosing and/or pharmacokinetics according to the Child-Pugh criteria, only 3 drugs had studies in patients with cancer [HCC for lenvatinib^14^; mixed tumor types (including ovarian cancer) for olaparib^15^^,^^16^; solid tumors for osimertinib^17^] (Table 1 and Figure 3). Moreover, these peer-reviewed studies were all limited in enrollment size (*N* ≤ 33 patients) (Table 1).

**Table 1:** Summary of peer-reviewed pharmacokinetic studies in patients with liver impairment assessed by Child-Pugh criteria

Drug	Indication	Study	Details
Alectinib	• For the treatment of patients with anaplastic lymphoma kinase-positive metastatic non-small-cell lung cancer	Morcos et al. *J Clin Pharmacol*. 2018;58:1618-1628.^18^	• Participant number: 28 (normal hepatic function, *n* = 12; Child-Pugh class B, *n* = 8; Child-Pugh class C, *n* = 8) • Patient characteristics: patients without cancer
Cabozantinib	• Patients with progressive metastatic medullary thyroid cancer • Patients with advanced renal cell carcinoma • Patients with hepatocellular carcinoma who had been previously treated with sorafenib^a^	Nguyen et al. *J Clin Pharmacol.* 2016;56:1130-1140.^19^	• Participant number: 26 (normal hepatic function, *n* = 10; Child-Pugh class A, *n* = 8; Child-Pugh class B, *n* = 8) • Patient characteristics: patients without cancer (not directly specified)
Dacomitinib	• First-line treatment of patients with metastatic non-small-cell lung cancer with epidermal growth factor receptor exon 19 deletion or exon 21 L858R substitution mutations	Giri et al. *Invest New Drugs*. 2015;33(4):931-941.^20^	• Participant number: 25 (normal hepatic function, *n* = 8; Child-Pugh class A, *n* = 8; Child-Pugh Class B, *n* = 9) • Patient characteristics: patients without cancer
Gilteritinib	• Patients who have relapsed or refractory acute myeloid leukemia with an FLT3 mutation	James et al. *Clin Pharmacokinet*. 2020 Oct;59(10):1273-1290.^21^	• Participant number: 24 (normal hepatic function, *n* = 8; Child-Pugh class B, *n* = 8; Child-Pugh class C, *n* = 8) • Patient characteristics: patients without liver cancer (other types of cancer not directly specified for exclusion)
Glasdegib	• In combination with low-dose cytarabine, for the treatment of newly diagnosed acute myeloid leukemia (AML) in adult patients who are ≥75 years old or who have comorbidities that preclude use of intensive induction chemotherapy	Masters et al. *Clin Pharmacol Drug Dev*. 2020 Dec 23.^22^	• Participant number: 24 (normal hepatic function, *n* = 8; Child-Pugh class B, *n* = 8; Child-Pugh class C, *n* = 8) • Patient characteristics: patients without cancer
Idelalisib	• Relapsed chronic lymphocytic leukemia, in combination with rituximab, in patients for whom rituximab alone would be considered appropriate therapy because of other comorbidities • Relapsed follicular B-cell non-Hodgkin’s lymphoma in patients who have received at least 2 prior systemic therapies • Relapsed small lymphocytic lymphoma in patients who have received at least 2 prior systemic therapies	Jin et al. *J Clin Pharmacol*. 2015;55:944-952.^23^	• Participant number: 32 (healthy matched control, *n* = 12; Child-Pugh class B, *n* = 10; Child-Pugh class C, *n* = 10) • Patient characteristics: patients without cancer (not directly specified)
Ivosidenib	• Adult patients with relapsed or refractory acute myeloid leukemia with a susceptible IDH1 mutation	Fan et al. *Clin Pharmacol Drug Dev*. 2021;10:99-109.^24^	• Participant number: 33 (normal hepatic function, *n* = 16; Child-Pugh class A, *n* = 9; Child-Pugh class B, *n* = 8) • Patient characteristics: patients without cancer
Lenvatinib	• For the treatment of patients with locally recurrent or metastatic, progressive, radioactive iodine-refractory differentiated thyroid cancer • In combination with everolimus, for the treatment of patients with advanced renal cell carcinoma following 1 prior antiangiogenic therapy • For the first-line treatment of patients with unresectable hepatocellular carcinoma	Shumaker et al. *J Clin Pharmacol*. 2015;55:317-327.^25^	• Participant number: 26 (normal hepatic function, *n* = 8; Child-Pugh class A, *n* = 6; Child-Pugh class B, *n* = 6; Child-Pugh class C, *n* = 6) • Patient characteristics: patients without cancer
		Ikeda et al. *Clin Cancer Res*. 2016;22:1385-1394.^14^	• Patient number: 20 patients (Child-Pugh class A, *n* = 9; Child-Pugh class B, *n* = 11) • Patient characteristics: patients with advanced hepatocellular carcinoma
Olaparib	• For the maintenance treatment of adult patients with recurrent epithelial ovarian, fallopian tube, or primary peritoneal cancer, who are in a complete or partial response to platinum-based chemotherapy • For the treatment of adult patients with deleterious or suspected deleterious germline *BRCA*-mutated advanced ovarian cancer who have been treated with 3 or more prior lines of chemotherapy	Rolfo et al. Pharmacokinetics and safety of olaparib in patients with advanced solid tumours and hepatic or renal impairment. In 18th Annual Meeting of the American Society for Clinical Pharmacology and Therapeutics. 13–18 March, 2017; Washington, DC. Abst PII–121.^16^ Pilla Reddy et al. *Clin Pharmacol Ther.* 2019;105:229-241.^15^	• Patient number: 23 patients (normal hepatic function, *n* = 13; Child-Pugh class A, *n* = 9; Child-Pugh class B results were ongoing at time of abstract publication) • Patient characteristics: major tumor types included ovarian (17%), breast (13%), and colon (13%) with chronic hepatic impairment where liver metastases were not the sole reason for any changes in liver function
Osimertinib	• For the treatment of patients with metastatic epidermal growth factor receptor T790M mutation positive non-small-cell lung cancer	Grande et al. *J Pharmacol Exp Ther*. 2019;369:291-299.^17^	• Patient number: 22 (normal hepatic function, *n* = 10; Child-Pugh class A, *n* = 7; Child-Pugh class B, *n* = 5) • Patient characteristics: patients with solid tumors and chronic hepatic impairment and where liver metastases were not the sole reason for any changes in liver function
Panobinostat	• For the treatment of patients with multiple myeloma in combination with bortezomib and dexamethasone; patient must have received ≥2 prior regimens, including bortezomib and an immunomodulatory agent	Slingerland et al. *Cancer Chemother Pharmacol*. 2014 Nov;74(5):1089-1098.^26^	• Patient number: 25 (normal hepatic function, *n* = 10; hepatic dysfunction: mild, *n* = 8; moderate, *n* = 6; severe, *n* = 1) • Patient characteristics: patients with advanced solid malignancies
Sonidegib	• For the treatment of adult patients with locally advanced basal cell carcinoma that has recurred following surgery or radiation therapy, or those who are not candidates for surgery or radiation therapy	Horsmans et al. *Clin Pharmacokinet*. 2018;57:345-354.^27^	• Participant number: 33 (normal hepatic function, *n* = 8; Child-Pugh Class A, *n* = 8; Child-Pugh class B, *n* = 8; Child-Pugh class C, *n* = 9) • Patient characteristics: patients without cancer (not directly specified)

a This indication occurred after the primary dates considered in this study (2014-2018).

## Conclusion

A variety of possible criteria may help clinicians assess liver function in patients with cancer.^1^^,^^6^^,^^28^ Based on our experience and the details from the reviewed protocols within this analysis, clinical trials of anticancer drugs have typically defined hepatic impairment—for the purposes of entry criteria and dose modifications—according to baseline liver function tests as opposed to Child-Pugh criteria (Supplementary Table S2, available at https://doi.org/10.1016/j.esmoop.2021.100162). Moreover, in routine oncological practice, liver function tests are utilized in relation to therapeutic decisions and drug dosing even though there is no consensus on which criteria should be used.^29^ Despite this routine use of liver function tests, the EMA and FDA have provided guidance for the use of the Child-Pugh criteria. As a result, among the oncologic, small molecule and cytotoxic drugs licensed from 2014 to 2018, the Child-Pugh criteria alone were cited in 38% of FDA prescribing information documents and in 63% of SmPCs for EMA-approved drugs (Supplementary Table S2, available at https://doi.org/10.1016/j.esmoop.2021.100162).

The number of FDA and EMA dosing recommendations based on Child-Pugh criteria alone is concerning, given that only 8% of drugs approved by the FDA from 2014 to 2018 had pivotal trials that used the Child-Pugh criteria within their inclusion and exclusion criteria. Moreover, only 42% (10/24) of drugs (that used Child-Pugh criteria for dosing recommendations in prescribing information and/or SmPCs) had a peer-reviewed publication describing dosing and pharmacokinetics according to Child-Pugh criteria, and all these studies were limited in size (≤33 patients). In addition, of these pharmacokinetics studies involving the Child-Pugh criteria only 3 out of these 10 recruited patients with cancer (Table 1; Figure 3).

Recently, Krens et al.^3^ put forward dosing recommendations for 160 anticancer drugs based on information derived from prescribing information and SmPCs issued by the FDA and EMA; recommendations for 26% of the reviewed drugs were made based on Child-Pugh score.^3^ We previously highlighted the issues surrounding these recommendations.^30^ Briefly, Child-Pugh criteria were historically developed to assess the prognosis of patients with chronic liver disease,^8^^,^^31^ and have never been validated for guiding dosing of anticancer drugs. The use of these criteria is further limited in patients with cancer because of the impact that the disease can have on the individual components of the criteria—thereby influencing a patient’s overall Child-Pugh score.^30^ As clear guidance is lacking about when the Child-Pugh criteria should be used in clinical practice, its application may be arbitrary and can lead to over- or under-dosing of cancer drugs. We propose that this widespread use of Child-Pugh criteria fails to address the most common clinical scenario encountered in oncological practice, namely appropriate dose selection in a patient with abnormal liver function secondary to metastatic involvement of the liver. Indeed, the minority of hepatic impairment pharmacokinetics studies that did enroll participants with cancer specifically required the liver impairment to be due to causes other than metastatic infiltration. Pharmacokinetic analyses with gefitinib help crystallize these issues because patients enrolled with moderate and severe hepatic impairment due to liver metastases had no clinically relevant differences in drug exposure; however, patients characterized as Child-Pugh class B and C secondary to cirrhosis had a significant increase in gefitinib exposure.^4^ These issues are especially concerning because it is unclear if similar inconsistencies exist with other anticancer treatments. The findings in our review further illustrate the lack of evidence for dosing recommendations based solely on Child-Pugh criteria.

The data in this review are limited by the publicly available information in study protocols and data that were accessible in peer-reviewed publications. Therefore, data that are held on file and not in the public domain are not represented. Moreover, updates to prescribing information and SmPCs may not be captured in this manuscript. Additionally, this review is not a systematic literature review, and therefore, the studies identified were limited by the results yielded from the selected search terms described and the authors’ knowledge of the field. A more systematic review in the future using searches in both PubMed and EMBASE may provide further clarity regarding the topics summarized in this manuscript. Despite these limitations, this review highlights a disconnect between the patient entry criteria used in pivotal studies and the criteria used to suggest drug dose modifications (which often use the upper limit of normal of transaminase and bilirubin levels) and the approved prescribing recommendations by the EMA and FDA regarding dosing in the presence of liver impairment (which often use Child-Pugh criteria). Based on the data within this review a discussion and reappraisal of the guidance contained within prescribing information and SmPCs relating to dose modification recommendations for patients with hepatic impairment is needed. Accepting that patients with severe liver disease will continue to be excluded from most pivotal studies, we nevertheless believe that SmPCs and prescribing information should more closely reflect the pivotal studies that lead to licensing of anticancer drugs when providing dosing guidance. Given the large number of patients and safety data that are accrued in pivotal studies, it may even be argued that it is detrimental to base clinical dosing on studies (with Child-Pugh criteria) that have limited evidence and are conducted in small groups of highly selected patients without cancer or with a different cancer type than that for which the drug is approved.

Based on the findings of this review, we suggest that the EMA and FDA review and critically appraise the appropriateness and validity of using Child-Pugh criteria in the context of anticancer drugs; moreover, all pivotal trial protocols should be published and available to guide clinical dosing decisions. In our opinion, the utilization of hepatic function entry criteria and dose modification criteria (which are based on the investigational brochure and contain the most updated pharmacokinetic data) of pivotal studies would provide a more logical and evidence-based approach to determine optimal dosing in patients with cancer and liver impairment.

## References

[1] Field K.M., Michael M. (2008). Part II: liver function in oncology: towards safer chemotherapy use. Lancet Oncol.

[2] Stuurman F.E., Nuijen B., Beijnen J.H., Schellens J.H. (2013). Oral anticancer drugs: mechanisms of low bioavailability and strategies for improvement. Clin Pharmacokinet.

[3] Krens S.D., Lassche G., Jansman F.G.A. (2019). Dose recommendations for anticancer drugs in patients with renal or hepatic impairment. Lancet Oncol.

[4] Horak J., White J., Harris A.L. (2011). The effect of different etiologies of hepatic impairment on the pharmacokinetics of gefitinib. Cancer Chemother Pharmacol.

[5] Hendrayana T., Wilmer A., Kurth V., Schmidt-Wolf I.G., Jaehde U. (2017). Anticancer dose adjustment for patients with renal and hepatic dysfunction: from scientific evidence to clinical application. Sci Pharm.

[6] European Medicines Agency, Committee for Medicinal Products for Human Use (CHMP) Guideline on the evaluation of the pharmacokinetics of medicinal products in patients with impaired hepatic function. http://www.ema.europa.eu/docs/en_GB/document_library/Scientific_guideline/2009/09/WC500003122.pdf.

[7] U.S. Department of Health and Human Services, Food and Drug Administration, Center for Drug Evaluation and Research (CDER), Center for Biologics Evaluation and Research (CBER) Guidance for Industry: pharmacokinetics in patients with impaired hepatic function: study design, data analysis, and impact on dosing and labeling. http://www.fda.gov/downloads/Drugs/GuidanceComplianceRegulatoryInformation/Guidances/ucm072123.pdf.

[8] Pugh R.N., Murray-Lyon I.M., Dawson J.L., Pietroni M.C., Williams R. (1973). Transection of the oesophagus for bleeding oesophageal varices. Br J Surg.

[9] Weersink R.A., Timmermans L., Monster-Simons M.H. (2019). Evaluation of information in summaries of product characteristics (SmPCs) on the use of a medicine in patients with hepatic impairment. Front Pharmacol.

[10] Elmeliegy M., Yang D.Z., Salama E., Parivar K., Wang D.D. (2021). Discordance between child-pugh and national cancer institute classifications for hepatic dysfunction: implications on dosing recommendations for oncology compounds. J Clin Pharmacol.

[11] Kudo M., Finn R.S., Qin S. (2018). Lenvatinib versus sorafenib in first-line treatment of patients with unresectable hepatocellular carcinoma: a randomised phase 3 non-inferiority trial. Lancet.

[12] Choueiri T.K., Escudier B., Powles T. (2015). Cabozantinib versus everolimus in advanced renal-cell carcinoma. N Engl J Med.

[13] Abou-Alfa G.K., Meyer T., Cheng A.L. (2018). Cabozantinib in patients with advanced and progressing hepatocellular carcinoma. N Engl J Med.

[14] Ikeda M., Okusaka T., Mitsunaga S. (2016). Safety and pharmacokinetics of lenvatinib in patients with advanced hepatocellular carcinoma. Clin Cancer Res.

[15] Pilla Reddy V., Bui K., Scarfe G. (2019). Physiologically based pharmacokinetic modeling for olaparib dosing recommendations: bridging formulations, drug interactions, and patient populations. Clin Pharmacol Ther.

[16] Rolfo C, de Vos-Geelen J, Isambert N, et al. Pharmacokinetics and safety of olaparib in patients with advanced solid tumours and hepatic or renal impairment. Presented at: 18th Annual Meeting of the American Society for Clinical Pharmacology and Therapeutics. March 13–18, 2017; Washington, DC. Abst PII–121.

[17] Grande E., Harvey R.D., You B. (2019). Pharmacokinetic study of osimertinib in cancer patients with mild or moderate hepatic impairment. J Pharmacol Exp Ther.

[18] Morcos P.N., Cleary Y., Sturm-Pellanda C. (2018). Effect of hepatic impairment on the pharmacokinetics of alectinib. J Clin Pharmacol.

[19] Nguyen L., Holland J., Ramies D. (2016). Effect of renal and hepatic impairment on the pharmacokinetics of cabozantinib. J Clin Pharmacol.

[20] Giri N., Masters J.C., Plotka A. (2015). Investigation of the impact of hepatic impairment on the pharmacokinetics of dacomitinib. Invest New Drugs.

[21] James A.J., Smith C.C., Litzow M. (2020). Pharmacokinetic profile of gilteritinib: a novel FLT-3 tyrosine kinase inhibitor. Clin Pharmacokinet.

[22] Masters J.C., LaBadie R.R., Salageanu J., Li J., Shaik N. (2020). Pharmacokinetics and safety of glasdegib in participants with moderate/severe hepatic impairment: a phase I, single-dose, matched case-control study. Clin Pharmacol Drug Dev.

[23] Jin F., Robeson M., Zhou H., Hisoire G., Ramanathan S. (2015). The pharmacokinetics and safety of idelalisib in subjects with moderate or severe hepatic impairment. J Clin Pharmacol.

[24] Fan B., Dai D., Cohen M. (2021). Effect of mild and moderate hepatic impairment on the pharmacokinetics, safety, and tolerability of a single dose of oral ivosidenib in otherwise healthy participants. Clin Pharmacol Drug Dev.

[25] Shumaker R., Aluri J., Fan J., Martinez G., Pentikis H., Ren M. (2015). Influence of hepatic impairment on lenvatinib pharmacokinetics following single-dose oral administration. J Clin Pharmacol.

[26] Slingerland M., Hess D., Clive S. (2014). A phase I, open-label, multicenter study to evaluate the pharmacokinetics and safety of oral panobinostat in patients with advanced solid tumors and various degrees of hepatic function. Cancer Chemother Pharmacol.

[27] Horsmans Y., Zhou J., Liudmila M. (2018). Effects of mild to severe hepatic impairment on the pharmacokinetics of sonidegib: a multicenter, open-label, parallel-group study. Clin Pharmacokinet.

[28] Grigorian A., O’Brien C.B. (2014). Hepatotoxicity secondary to chemotherapy. J Clin Transl Hepatol.

[29] Field K.M., Dow C., Michael M. (2008). Part I: liver function in oncology: biochemistry and beyond. Lancet Oncol.

[30] Palmieri C., Macpherson I. (2019). Use of the Child-Pugh score in anticancer drug dosing decision making: proceed with caution. Lancet Oncol.

[31] Child C.G., Turcotte J.G., Child C.G. (1964). Surgery and portal hypertension. Liver and Portal Hypertension.

