# Supplementary material for: A review of the evidence base for utilizing Child-Pugh criteria for guiding dosing of anticancer drugs in patients with cancer and liver impairment
Source: ESMO Open. 2021 Jun 5;6(3):100162. doi: 10.1016/j.esmoop.2021.100162 (PMC8190488; doi:10.1016/j.esmoop.2021.100162)
Supplement: Supplementary Tables [file mmc1.docx]

**SUPPLEMENTAL MATERIAL**

**Supplemental Table 1.** Child-Pugh classification

| **Child-Pugh Scores** | | | |
| --- | --- | --- | --- |
|  | **1 point** | **2 points** | **3 points** |
| **Total bilirubin, mg/dL** | < 2 | 2–3 | > 3 |
| **Albumin, g/dL** | > 3.5 | 2.8–3.5 | < 2.8 |
| **Prothrombin time, seconds prolonged** | < 4 | 4–6 | > 6 |
| **Ascites** | Absent | Slight | Moderate |
| **Encephalopathy, grade** | None | 1 or 2 | 3 or 4 |

Class A (mild) = 5–6 points; class B (moderate) = 7–9 points; class C (severe) = 10–15 points.

**Supplemental Table 2.** Use of liver-function tests and Child-Pugh score for approved oncologic treatments

| **Drug** | **Dosing Guidance in Patients With Hepatic Impairment** | **Inclusion/Exclusion Criteria Based on Liver Function/Disease Data From Pivotal Studies** | **Protocol Available With Publication?** | **Available Child-Pugh Score Data (PI, SmPCs, PubMed search)** |
| --- | --- | --- | --- | --- |
| Abemaciclib | **FDA; PI/EMA; SmPC**   - **Child-Pugh classes A and B**: No dose adjustments are necessary - **Child-Pugh class C**: Decrease dosing frequency to once daily | **MONARCH-1: *Dickler et al. Clin Cancer Res. 2017;23:5218-24.***[1]  **Inclusion criteria**   - Adequate organ function   **MONARCH-2: *Sledge et al. J Clin Oncol. 2017;35:2875-84.***[2]  **Inclusion criteria**   - Bilirubin ≤ 1.5 x ULN - ALT ≤ 3.0 x ULN   **Exclusion criteria**  **•** Active or detectable viral infection  **MONARCH-3: *Goetz et al. J Clin Oncol. 2017;35:3638-46.***[3]  **Inclusion criteria**   - Bilirubin ≤ 1.5 x ULN - ALT and AST ≤ 3.0 x ULN (≤ 5.0 x ULN if liver metastases  are present)   **Exclusion criteria**   - Active or detectable viral infection | No  Yes  Yes | **FDA; PI**  **Study details**   - Single 200-mg oral dose - No data on underlying cause of hepatic impairment   **Patients: Results**   - **Normal hepatic function (n=10)** - **Child-Pugh class A (n = 9):** AUC_0-INF_ of abemaciclib plus its active metabolites (M2, M18, M20) increased 1.2-fold - **Child-Pugh class B (n = 10):** AUC_0-INF_ increased 1.1-fold - **Child-Pugh class C (n = 6):** AUC_0-INF_ increased 2.4-fold and half-life increased from 24 to 55 hours |
| Acalabrutinib | **FDA; PI**   - **Mild or moderate hepatic impairment (total bilirubin ≤ ULN and AST > ULN, or total bilirubin greater than ULN and any AST; Child Pugh Classes B & C)^a^:** No clinically relevant PK difference was observed in patients with mild or moderate hepatic impairment; dose modifications not required. **Severe hepatic impairment (Child-Pugh Class C)^b^:** Safety not studied; avoid administration   ***^a,b^Definitions not specified in ‘Recommended Dosage for Hepatic Impairment’, but inferred, since both liver-function tests and Child-Pugh is referenced*** | **ACE-LY-004:** ***Wang et al. Lancet. 2018;391(10121):659-67.***[4]  **Exclusion criteria**   - Known history of HIV, or active infection with HCV, HBV, or any uncontrolled systemic infection | No | **FDA; PI**  **Study details**   - Dose not reported - No data on underlying cause of hepatic impairment   **Patients: Results**   - **Normal hepatic function** - **Child-Pugh class A:**   AUC increased 1.9-fold   - **Child-Pugh class B:**   AUC increased 1.5-fold   - **Child-Pugh class C:**   AUC increased 5.3-fold |
| Alectinib | **FDA; PI /EMA; SmPC**   - **Child-Pugh classes A and B:** No starting-dose adjustment required - **Child-Pugh class C**: Starting dose of 450 mg taken twice daily with food (total daily dose of 900 mg) | **NP28673: *Ou et al. J Clin Oncol. 2016;34:661-8.***[5]  **Inclusion criteria**   - Bilirubin < 2.0 mg/dL - AST and ALT < 2.5 times or less ULN   **Exclusion criteria**   - Active or uncontrolled infectious diseases requiring treatment   **NP28761: *Shaw et al. Lancet Oncol. 2016;17:234-24.***[6]  **Inclusion criteria**   - AST and ALT ≤ 2.5 x ULN (≤ 5.0 x ULN in patients with liver metastases)   **Exclusion criteria**   - History of or current active infection with HBV, HBC, or HIV   **ALEX study: *Peters et al. N Engl J Med. 2017;377:829-38.***[7]  **Exclusion criteria**  Liver disease characterized by:   - ALT or AST > 3.0 x ULN (≥ 5.0 x ULN for patients with concurrent liver metastasis) confirmed on 2 consecutive measurements *OR* - Impaired excretory function (eg, hyperbilirubinemia) or synthetic function or other conditions of decompensated liver disease, such as coagulopathy, hepatic encephalopathy, hypoalbuminemia, ascites, and bleeding from esophageal varices *OR* - Acute viral or active autoimmune, alcoholic, or other types of hepatitis | No  No  Yes | ***Morcos et al. J Clin Pharmacol.2018;58:*** ***1618-1628.***[8]  **Study details**   - Single 300-mg dose - Patients without hepatocellular carcinoma or acute liver disease - No details of underlying hepatic pathology   **Patients: Results**   - **Healthy subjects (n = 12)** - **Child-Pugh class B (n = 8):** AUC_0-inf_ GMR: 160% (90% CI: 105–243) - **Child-Pugh class C (n = 8):** AUC_0-inf_ GMR: 220% (90% CI: 131–369) |
| Apalutamide | **FDA; PI**   - No dosing guidance provided for patients with underlying hepatic impairment (Child-Pugh classes A, B and C)^a^   ***^a,^Definition not specified in ‘Recommended Dosage for Hepatic Impairment’, but inferred, since Child-Pugh is referenced***  **EMA guidance**   - **Mild to moderate (Child-Pugh classes A and B):** No dose adjustment required - **Severe hepatic impairment (Child-Pugh Class C):** No data available; not recommended for use | **SPARTAN:** ***Smith et al. N Engl J Med. 2018;378:1408-18.***[9]  **Inclusion criteria**  Adequate organ function, as defined by:   - Serum AST and ALT ≤ 2.5 x ULN - Total serum bilirubin ≤ 1.5 x ULN     **Exclusion criteria**   - Active infection, such as HIV | Yes | **EMA; SmPC**  **Study details**   - Single oral 240-mg dose - No data on underlying cause of hepatic impairment   **Patients: Results**   - **Normal hepatic function (n = 8)** - **Child-Pugh class A (n = 8):**  GMRs for AUC and C_max_ were 95% and 102%, respectively - **Child-Pugh class B (n = 8):**   GMRs for AUC and C_max_ were 113% and 104%, respectively |
| Belinostat | **FDA; PI^a^**   - **Moderate and severe hepatic impairment**: Not studied; insufficient data to recommend a dose   ***^a^Patients with moderate and severe hepatic impairment: (total bilirubin > 1***.***5 x ULN) were excluded from clinical trials*** | **BELIEF (CLN-19) Study: *O’Connor et al. J Clin Oncol. 2015;33:2492-9****.*[10]  **Inclusion criteria**   - Total bilirubin ≤ 1.5 x ULN, or ≤ 3.0 x ULN if documented hepatic involvement with lymphoma, or ≤ 5.0 x ULN if history of Gilbert’s disease - AST and ALT ≤ 2.5 x ULN (≤ 5.0 x ULN limit if documented hepatic involvement with lymphoma)   **Exclusion criteria**   - Any known active infection | No |  |
| Binimetinib  (trials include both encorafenib and binimetinib; these drugs are indicated in combination) | **FDA; PI**   - **Mild hepatic impairment (total bilirubin > 1.0 and ≤ 1**.**5 × ULN and any AST or total bilirubin ≤ ULN and AST > ULN):** No dose adjustment is required - **Moderate (total bilirubin > 1**.**5 and  ≤ 3**.**0 × ULN and any AST) or severe (total bilirubin levels > 3.0 × ULN and any AST) hepatic impairment:** Reduce dose to 30 mg twice daily   **EMA; SmPC**   - **Child-Pugh class A**: No dose adjustments are required - **Child-Pugh classes B and C**: Not recommended for use because encorafenib is not recommended for use in these patients | **CMEK162X2110: *Data not yet published***  **Exclusion criteria**   - Abnormal laboratory values at screening/baseline (values not specified)   **LOGIC-2 (CLGX818X2109): *Data not yet published***  **Exclusion criteria**  Any of the following laboratory values at screening/baseline:   - AST/SGOT or ALT/SGPT > 2.5 x ULN, or > 5.0 x ULN if liver metastases are present - Serum total bilirubin > 1.5 x ULN   **COLOMBUS (CMEK162B2301):** ***Dummer et al. Lancet Oncol. 2018;603-15***.[11]  **Exclusion criteria**   - HIV positive, or active HBV and/or HCV | No | **EMA; SmPC**  **Study details**   - Dose not reported - No data on underlying cause of hepatic impairment   **Patients: Results**   - **Child-Pugh class A**:   Similar exposure to subjects with normal liver function   - **Child-Pugh classes B and C:**   A 2-fold increase in total binimetinib exposure (AUC) and 3-fold increase in unbound binimetinib exposure |
| Brigatinib | **FDA; PI**   - **Child-Pugh classes A and B:** No dose adjustments are required - **Child-Pugh class C:** Reduce once daily dose by approximately 40% (ie, from 180 mg to 120 mg, 120 mg to 90 mg, or from 90 mg to 60 mg)   **EMA; SmPC**   - **Child-Pugh classes A and B**: No dose adjustments are necessary - **Child-Pugh class C**: Reduce the starting dose to 60 mg once daily for the first 7 days, then 120 mg once daily thereafter | **ALTA:** ***Kim et al. J Clin Oncol. 2017;35:2490-8.***[12]  **Inclusion criteria**  Have adequate organ function, as determined by:   - ALT/AST ≤ 2.5 x ULN (≤ 5.0 x ULN is acceptable if liver metastases are present) - Total serum bilirubin ≤ 1.5 x ULN (< 3.0 x ULN for patients with Gilbert’s syndrome)   Exclusion criteria   - HIV positive or active infection | Yes | **FDA; EMA**  **Study details**   - Single 90-mg dose *OR* - 180 mg once daily following a  7-day lead-in at 90 mg once daily - No data on underlying cause of hepatic impairment   **Patients: Results**   - **Normal hepatic function (n = 9)** - **Child-Pugh class A (n = 6):** PK similar to healthy subjects - **Child-Pugh class B (n = 6):** PK similar to healthy subjects - **Child-Pugh class C (n = 6):** Unbound AUC_0-INF_ was 37% higher |
| Cabozantinib  Commercially available in 2 formulations: Cabometyx®  and  Cometriq®; Cometriq® was approved in 2012 and is therefore excluded from analysis | **FDA; PI for Cabometyx** *RCC and HCC*   - **Mild hepatic impairment (total bilirubin ≤ ULN and AST > ULN or total bilirubin > 1 to 1.5x ULN and any AST value)^a^ :**: No guidance provided - **Child-Pugh class B**: Reduce starting dose to 40 mg once daily - **Child-Pugh class C:** Avoid use   **EMA; SmPC for Cabometyx** *RCC and HCC*   - **Child-Pugh class A**: No dose adjustment needed - **Child-Pugh class B**: Limited data, therefore no dosing recommendation - **Child-Pugh class C**: No clinical data, therefore not recommended for use   **^a^Definition not provided within dosing but noted within the PI in ‘Specific Populations’.** | **RCC-METEOR Trial: *Choueiri et al. N Engl J Med. 2015;373:1814-23.***[13]  **Inclusion criteria**   - Adequate organ function: ALT and AST < 3.0 x ULN - Total bilirubin ≤ 1.5 x ULN. For subjects with Gilbert’s disease  ≤ 3.0 mg/dL (51.3 μmol/L)   **Exclusion criteria**   - Moderate to severe hepatic impairment (Child-Pugh class B  or C) - Active infection requiring systemic treatment, HIV, or chronic HBV or HCV   **RCC-CABOSUN/ Alliance A031203: *Choueiri et al. J Clin Oncol. 2017;35:591-7.***[14]  **Inclusion criteria**  Required initial laboratory values:   - Total bilirubin, ≤ 1.5 x ULN - AST/ALT ≤ 2.5 x ULN   **HCC-CELESTIAL Trial*: Abou-Alfa et al. N Engl J Med. 2018;379:54-63.***[15]  **Inclusion criteria**   - Child-Pugh class A - Total bilirubin ≤ 2 mg/dL (≤ 34.2 μmol/L) within 7 days before randomization - Serum albumin ≥ 2.8 g/dL (≥ 28 g/L) within 7 days before randomization - ALT and AST < 5.0 x ULN within 7 days before randomization - Antiviral therapy per local standard of care if active HBV infection | Yes  Yes  Yes | ***Nguyen et al. J Clin Pharmacol. 2016;56:1130-40.***[16]  **Study details**   - Single oral 60-mg dose - All men - No data on underlying cause of hepatic impairment   **Patients: Results**   - **Normal hepatic function (n = 10)** - **Child-Pugh class A (n = 8)***:*   AUC_0-inf_ GMR: 181%  (90% CI: 121–270)   - **Child-Pugh class B (n = 8)***:*   AUC_0-inf_ GMR: 163%  (90% CI: 107–247) |
| Ceritinib | **FDA; PI**   - **Child-Pugh classes A and B**: No dose adjustment necessary - **Child-Pugh class C:** Reduce dose by approximately one-third, rounded to the nearest multiple of the 150-mg dosage strength   **EMA; SmPC**   - **Mild or moderate hepatic impairment (Child-Pugh classes A and B)^a^**: No dose adjustment necessary - **Severe hepatic impairment (Child-Pugh Class C)^a^**: Reduce dose by approximately one-third (rounded to the nearest multiple of 150-mg dosage strength)   ***^a^Definition not given within dosing, but inferred from information within the SmPC, since, Child-Pugh scores is referenced*** | **ASCEND 1 Trial: *Shaw et al. N Engl J Med. 2014;370;13:1189-97.***[17]    **Inclusion criteria**   - Serum total bilirubin ≤ 1.5 x ULN - Alkaline phosphatase, AST and ALT ≤ 2.5 x ULN, except for patients with tumor involvement of the liver who must have AST and ALT ≤ 5.0 x ULN   **Exclusion criteria**   - Acute or chronic liver disease - Evidence of previous hepatitis viral infection such as HBV or HCV   **ASCEND 4 Trial: *Soria et al. Lancet. 2017;389:917-29*.**[18]  **Inclusion criteria**   - Total bilirubin < 1.5 x ULN except for patients with Gilbert’s syndrome who may be included only if total bilirubin < 3.0 x ULN or direct bilirubin < 1.5 x ULN - Aspartate transaminase (AST) < 2.5 x ULN, except for patients with liver metastasis, who are included only if AST < 5.0 x ULN - ALT < 2.5 x ULN, except for patients with liver metastasis, who are only included if ALT < 5.0 x ULN - ALP < 5.0 x ULN   **ASCEND-5 Trial: *Shaw et al. Lancet Oncol. 2017;18:874-86.***[19]  **Inclusion criteria**   - Total bilirubin < 1.5 × ULN, except for patients with Gilbert’s syndrome, who were included only if total bilirubin < 3.0 × ULN or direct bilirubin < 1.5 × ULN - AST < 2.5 × ULN, except for patients with liver metastasis, who were included only if AST < 5.0 × ULN - ALT < 2.5 x ULN, except for patients with liver metastasis, who were included only if AST < 5.0 × ULN - ALP < 5.0 × ULN | Yes  Yes  No | **EMA; SmPC**  **Study details**   - Single 750-mg dose under fasting conditions - No information on underlying cause of hepatic impairment   **Patients: Results**   - **Healthy (n = 8)** - **Child-Pugh class A (n = 8):**  Geometric mean AUC_inf_ (unbound AUC_inf_) increased by 18% (35%) - **Child-Pugh class B (n = 7):**  Geometric mean AUC_inf_ (unbound AUC_inf_) increased by 2% (22%) - **Child-Pugh class C (n = 7):**  Geometric mean AUC_inf_ (unbound AUC_-inf_) increased by 66% (108%) |
| Cobimetinib | **FDA; PI**   - **Child-Pugh classes A, B, and C**: No adjustment in the starting dose required   **EMA; SmPC**   - No dose adjustments are recommended for patients with hepatic impairment (Child-Pugh classes A, B and C)^a^ - Severe hepatic impairment may cause increased plasma concentrations of unbound cobimetinib compared with normal hepatic function.   ***^a^Definition not specified in dosing, but Inferred from information within the SmPC since, Child-Pugh class is referenced*** | **BRIM7: *Ribas et al. Lancet Oncol. 2014;15:954-65.***[20]  **Inclusion criteria**  Adequate hepatic function, defined by:   - Albumin ≥ 2.5 g/dL - Bilirubin ≤ 1.5 x ULN - AST, ALT, and ALP ≤ 2.5 x ULN, with the following exceptions:   - Patients with document liver metastases: AST and/or ALT ≤ 5.0 x ULN   - Patients with documented liver or bone metastases: ALP ≤ 5.0 x ULN   **coBRIM: *Larkin et al. N Engl J Med. 2014;371:1867-76.***[21]  **Inclusion criteria**  Adequate hepatic function, defined by the following laboratory results obtained within 14 days prior to first dose of study-drug treatment:   - Albumin ≥ 2.5 g/dL - Bilirubin ≤ 1.5 x ULN - AST, ALT, and ALP ≤ 3.0 x ULN with the following exceptions:   - Patients with documented liver metastases: AST and/or ALT ≤ 5.0 x ULN   - Patients with documented liver or bone metastases: ALP ≤ 5.0 x ULN | Yes  Yes | **EMA; SmPC**  **Study details**   - Single 10-mg dose - No data on underlying cause of hepatic impairment   **Patients: Results**   - **Healthy subjects (n = 10)** - **Child-Pugh class A (n = 6) and Child-Pugh class B (n = 6)**   Total and unbound cobimetinib exposure was similar to healthy subjects   - **Child-Pugh class C (n = 10)**   AUC_0-inf_ GMR: 0.69 compared to healthy subjects. Unbound cobimetinib exposures approximately 2-fold higher than healthy subjects |
| Copanlisib | **FDA; PI**   - **Mild hepatic impairment (bilirubin ≤1 x ULN and AST > ULN, or total bilirubin > 1.0 to 1.5 x ULN and any AST)^a^** No dose adjustment is necessary - **Moderate hepatic impairment (Child-Pugh class B):** Reduce dose to 45 mg - **Severe hepatic impairment (Child-Pugh class C)^a^:** Not studied; no guidance provided   ***^a,^Definition not specified in ‘Recommended Dosage for Hepatic Impairment’, but noted within the PI under ‘Use in specific populations’*** | **CHRONOS-1: *Dreyling et al. Ann Oncol. 2017;28:2169-78.***[22]  **Inclusion criteria**   - Adequate hepatic function, as assessed within 7 days before starting study treatment   **Exclusion criteria**   - HBV or HCV | No | **FDA; PI**  **Study details**   - Single IV dose of 12-mg - No data on underlying cause of hepatic impairment   **Patients: Results**   - **Normal hepatic function** - **Child-Pugh class B:**   Geometric mean of total copanlisib C_max_ increased 1.38-fold; geometric mean of total copanlisib AUC increased 1.71-fold   - **Child-Pugh class C:**   PK of copanlisib is unknown |
| Dacomitinib | **FDA; PI**   - **Mild (total bilirubin ≤ ULN with AST > ULN or total bilirubin > 1 to 1.5 x ULN with any AST):** No dose adjustment required - **Moderate (total bilirubin > 1.5 to 3 x ULN and any AST) hepatic impairment:** No dose adjustment required - **Severe hepatic impairment (total bilirubin > 3 to 10 x ULN and any AST):** Recommended dose has not been established   **EMA; SmPC**   - **Child-Pugh classes A and B:** no dose adjustments required - **Child-Pugh class C:** No data; treatment not recommended in this population | **ARCHER 1050 Trial: *Wu et al. Lancet Oncol. 2017;18:1454-66****.*[23]  **Inclusion criteria**  Adequate hepatic function, as defined by:   - Bilirubin ≤ 1.5 x ULN - AST or ALT ≤ 2.5 x ULN (≤ 5.0 x ULN if hepatic metastases are present) | Yes | ***Giri et al. Invest New Drugs. 2015;33(4):931-941.***[24]  **Study details**   - Single 30-mg dose - All male - Hepatic dysfunction due to hepatocellular disease (hepatic carcinoma and hepatorenal syndrome excluded)   **Patients: Results**   - **Normal hepatic function (n = 8)** - **Child-Pugh class A (n = 8):** AUC_inf_ and C_max_ similar to healthy patients - **Child-Pugh class B (n=9):** AUC_inf_ and C_max_ decreased by 15% and 20% |
| Daunorubicin and cytarabine encapsulated in liposomes | **FDA; PI**   - **Bilirubin ≤ 3 mg/dL**: No dose adjustments necessary - **Bilirubin > 3 mg/dL**: Not studied; no guidance provided   **EMA; SmPC**   - **Bilirubin** ≤ **50 µmol/L:** No dose adjustments necessary - **Bilirubin > 50 µmol/L**: No clinical experience; use in patients with severe hepatic impairment only if the benefits outweigh the risks | **CTLR0310-301: *Lancet et al. J Clin Oncol. 2018;36:2684-92.***[25]  **Inclusion criteria**   - Serum total bilirubin < 2.0 mg/dL - Serum ALT or AST < 3 x ULN   **Exclusion criteria**   - Known HIV or evidence of active HBV or HCV infection | Yes | **N/A** |
| Duvelisib | **FDA; PI**  **Hepatic impairment (Child-Pugh Class A, B and C)^a^: had no clinically significant effect on exposure of duvelisib;** no specific dosing guidance was provided  ***^a^Definition not given within ‘Indications and Usage’, but inferred, since Child-Pugh class was used to define hepatic impairment in ‘specific populations’*** | **DUO: *Flinn et al. Blood 2018;132:2446-55.***[26]  **Inclusion criteria**  Adequate hepatic function, as defined by:   - AST and ALT ≤ 3 x ULN - Total bilirubin ≤ 1.5 x ULN   **Exclusion criteria**   - HIV infection, and prior, current, or chronic HBV or HCV - History of alcohol abuse or chronic liver disease (other than metastatic disease to the liver)   **DYNAMO: *Flinn et al. J Clin Oncol. 2019;37:912-922.***[27]  **Inclusion criteria**  Adequate hepatic function, as defined by:   - AST and ALT ≤ 3 x ULN - Total bilirubin ≤ 1.5 x ULN   **Exclusion criteria**   - Prior, current, or chronic HIV, HBV, or HCV | Yes  No | **FDA; PI**  **Study details**   - Dose not reported - No data on underlying cause of hepatic impairment   **Patients: Results**   - **Child-Pugh classes A, B, and C**   No clinically significant effect on exposure of duvelisib |
| Enasidenib | **FDA; PI**   - **No dosing guidance provided for patients with underlying hepatic impairment (mild,** **defined**   **as total bilirubin ≤ ULN and AST >ULN or total bilirubin 1 to 1.5 x ULN and any AST)^a^**  ***^a^Definition not given within dosing, but inferred, since, liver-function tests were used to define hepatic function in ‘Specific Populations’*** | **AG221-C-001:** ***Stein et al. Blood. 2017;130:722-31.***[28]  **Inclusion criteria**  Adequate hepatic function, as defined by:   - Serum total bilirubin ≤ 1.5 x ULN, unless considered due to Gilbert’s disease, a gene mutation in UGT1A1, or leukemic organ involvement - AST, ALT, and ALP ≤ 3.0 x ULN unless considered due to leukemic organ involvement   **Exclusion criteria**   - Known infection with HIV or active HBV or HCV | No | **N/A** |
| Encorafenib  (trials include both encorafenib and binimetinib; these drugs are indicated in combination) | **FDA; PI**   - **Child-Pugh class A:** No dose adjustments necessary - **Child-Pugh classes B and C:** A recommended dose has not been established   **EMA; SmPC**   - **Child-Pugh class A**: Administer with caution; reduce starting dose to  300 mg once daily - **Child-Pugh classes B and C**: No clinical data; Not recommended for use | **See pivotal studies listed for binimetinib.** |  | **EMA; SmPC**  **Study details**   - No details on dose - No data on underlying cause of hepatic impairment   **Patients: Results**   - **Normal hepatic function** - **Child-Pugh class A:**   A 25% increase in total encorafenib exposure and 55% increase in unbound encorafenib exposure   - **Child-Pugh classes B and C:**   Not evaluated |
| Gilteritinib | **FDA; PI**   - **Hepatic impairment (Child-Pugh classes A, B and C)^a^:** had no meaningful clinical effects on PK; no dosing guidance provided   ***^a^Defintion not given within dosing, but inferred, since Child-Pugh Class was used to define hepatic function in ‘Specific Populations’***  **EMA guidance**   - **Child-Pugh classes A and B:** No dose adjustments necessary - **Child-Pugh class C**: Safety unknown; not recommended for use | **ADMIRAL (2215-CL-0301): *Perl et al. N Engl J Med. 2019;381:1728-1740.***[29]  **Inclusion criteria**   - Serum AST and ALT ≤ 2.5 x ULN - Serum total bilirubin ≤ 1.5 x ULN   **Exclusion criteria**   - Known HIV, or active HBV, HCV, or other hepatic disorder | Yes | **FDA; PI**  **Study details**   - Dose not reported - No data on underlying cause of hepatic impairment   **Patients: Results**   - **Child-Pugh classes A and B:**   No clinically meaningful effects on PK of gilteritinib   - **Child-Pugh class C:**   Effect on PK unknown |
| Glasdegib | **FDA; PI**   - **Mild [total bilirubin ≤ ULN and AST > ULN or total bilirubin 1–1.5 × ULN and any AST]; moderate and severe [Child-Pugh classes B and C]^a^:** No dosing guidance provided   ***^a^Definitions not given within dosing, but inferred, since both liver function tests, and Child-Pugh Class is used to define hepatic impairment in ‘specific populations’*** | **BRIGHT AML 1003**: ***Cortes et al. Leukemia. 2019;33:379-89.***[30]  **Inclusion criteria**   - Serum AST and ALT ≤ 3 x ULN, or ≤ 5 x ULN if liver function abnormalities are due to underlying malignancy - Total serum bilirubin ≤ 2 x ULN (unless documented Gilbert’s syndrome) | No | **FDA; PI**  **Study details**   - Single 100-mg dose - No data on underlying cause of hepatic impairment   **Patients: Results**   - **Normal hepatic function** - **Child-Pugh class B:**   AUC_0-inf_ increased by 11%   - **Child-Pugh class C:**   AUC_0-inf_ decreased by 24% |
| Idelalisib | **FDA; PI**   - **ALT or AST or bilirubin > ULN**: No dose adjustments necessary - **AST or ALT > 2**.**5 x ULN or bilirubin > 1**.**5 x ULN:** Limited clinical data; monitor subjects with baseline hepatic impairment for signs of toxicity   **EMA; SmPC**   - **Mild or moderate hepatic impairment**: **(Child-Pugh classes A, B and C)^a^:** No dose adjustments necessary, but an intensified monitoring of adverse reactions recommended - **Severe hepatic impairment (Child-Pugh class C)^a^:** Insufficient data to make a dose a recommendation; use caution   ***^a^Definition not specified in dosing section, but inferred from information within the SmPC, because Child-Pugh Class is referenced*** | **DELTA (101-09): *Gopal et al. N Engl J Med. 2014;370:1008-18*.**[31]  **Inclusion criteria**  Required baseline laboratory values (within 2 weeks prior to start of study) as follows:   - Serum total bilirubin ≤ 1.5 x ULN (unless elevated due to Gilbert’s syndrome) - Serum ALT and AST ≤ 2.5 x ULN - Negative antibodies for HIV, negative HBsAG for HBV, and negative viral RNA for HCV   **Exclusion criteria**   - Known history of drug-induced liver injury, chronic active HCV, chronic active HBV, alcoholic liver disease, nonalcoholic steatohepatitis, primary biliary cirrhosis, ongoing extrahepatic obstruction caused by stones, cirrhosis of the liver, or portal hypertension   **GS-US-312-0116: *Furman et al. N Engl J Med. 2014;370:997-1007*.**[32]  **Inclusion criteria**  Required baseline laboratory values (within 4 weeks prior to randomization) as follows:   - Serum total bilirubin ≤ 1.5 x ULN (unless elevated due to Gilbert’s syndrome) - Serum ALT and AST ≤ 2.5 x ULN   Negative antibodies for HIV, negative HBsAG and negative HBc antibody for HBV, and negative viral RNA for HCV  **Exclusion criteria**   - Known history of drug-induced liver injury, chronic active HCV, chronic active HBV, alcoholic liver disease, non-alcoholic steatohepatitis, primary biliary cirrhosis, ongoing extrahepatic obstruction caused by cholelithiasis, cirrhosis of the liver, or portal hypertension   **GS-US-312-0119: *Jones et al. Lancet Haematol. 2017;4(3):E114-126*.**[33]  **Inclusion criteria**  Required baseline laboratory values (within 4 weeks prior to randomization) as follows:   - Serum total bilirubin ≤ 1.5 x ULN (unless elevated due to Gilbert’s syndrome or hemolysis) - Serum AST and ALT ≤ 2.5 x ULN - Negative antibodies for HIV, negative HBsAg and negative HBc antibody for HBV (or positive HBc and negative HBV DNA by quantitative PCR), and negative viral RNA for HCV   **Exclusion criteria**   - Ongoing drug-induced liver injury, chronic active HPC, chronic active HBV, alcoholic liver disease, non-alcoholic steatohepatitis, primary biliary cirrhosis, extrahepatic obstruction caused by cholelithiasis, cirrhosis of the liver, or portal hypertension | Yes  Yes  Yes | **Jin et al. *J Clin Pharmacol.* 2015;55:944–52*.***[34]  **Study details**   - Single oral 150-mg dose - No data on underlying cause of hepatic impairment   **Patients: Results**   - **Normal hepatic function** **(n = 12)** - **Child-Pugh class B (n = 10)**:  AUC_0-inf_ 158% (90% CI: 125–199%) - **Child-Pugh class C** **(n = 10):**  AUC_0-inf_ 159% (90% CI: 121–208%) - Changes are not considered clinically relevant |
| Irinotecan in a pegylated liposomal formulation | **FDA; PI**  **No dosing guidance provided for patients with underlying hepatic impairment (bilirubin 1-2 mg/dL or elevated ALT/AST)^a^**  **^a^Criteria not specified in dosing but noted under ‘Specific populations’**  **EMA; SmPC**   - **Bilirubin > 2.0 mg/dL, or AST and ALT > 2.5 x ULN (> 5** **x ULN if liver metastasis is present):** Avoid administration | **NAPOLI-1: *Wang-Gillam et al. Lancet. 2016;387:545-57.***[35]  **Inclusion criteria**  Adequate hepatic function, as defined by:   - Normal serum total bilirubin according to local institutional standards - Albumin levels ≥ 30 g/L | No | **N/A** |
| Ivosidenib | **FDA; PI**   - **Child-Pugh classes A and B:** No dose adjustments necessary - **Child-Pugh class C:** Not studied; consider risks and potential benefits before initiating treatment | **AG120-C-001: *DiNardo et al. N Engl J Med. 2018;378:2386-98.***[36]  **Inclusion criteria**  Adequate hepatic function, as defined by:   - AST, ALT, and ALP ≤ 3.0 x ULN, unless considered due to leukemic involvement - Serum total bilirubin ≤ 1.5 x ULN, unless considered due to Gilbert’s disease or leukemic involvement   **Exclusion criteria**   - Known infection with HIV, or active HBV or HCV | Yes | **FDA; PI**  **Study details**   - Single 500-mg dose - No data on cause of underlying hepatic impairment   **Patients: Results**   - **Normal hepatic function** - **Child-Pugh class A:**   AUC_0-inf_ GMR (90% CI): 0.85 (0.62–1.15)   - **Child-Pugh class B:**   AUC_0-inf_ GMR (90% CI): 0.71 (0.48–1.05)   - **Child-Pugh class C**:   Effect on PK unknown |
| Ixazomib | **FDA; PI**   - **Moderate hepatic impairment (total bilirubin > 1**.**5 to 3 x ULN):** Reduce starting dose to 3 mg daily - **Severe hepatic impairment (total bilirubin > 3 x ULN):** Reduce starting dose to 3 mg daily   **EMA; SmPC**   - **Mild hepatic impairment** **(bilirubin ≤ ULN and AST > ULN, or total bilirubin > 1 to 1.5 x ULN and any AST):** No dose adjustments necessary - **Moderate hepatic impairment** **(total bilirubin > 1.5 to 3 x ULN):** Reduce starting dose to 3 mg daily - **Severe hepatic impairment** **(total bilirubin > 3 x ULN):** Reduce starting dose to 3 mg daily | **C16010: *Moreau et al. N Engl J Med. 2016;374:1621-34.***[37]  **Inclusion criteria**   - Total bilirubin ≤ 1.5 x ULN - ALT and AST ≤ 3 x ULN   **Exclusion criteria**   - Ongoing or active systemic infection, active HBV infection, active HCV infection, or known HIV positive | Yes | **N/A** |
| Larotrectinib | **FDA; PI**   - **Child-Pugh class A**: No dose adjustments necessary - **Child-Pugh classes B and C**: Reduce starting dose by 50%   **EMA; SmPC**   - **Child-Pugh class A:** No dose adjustments necessary - **Child-Pugh classes B and C:** Reduce starting dose by 50% | **LOXO-TRK-14001: *Hong et al. Ann Oncol. 2019;30:325-31.***[38]  **Inclusion criteria**   - ALT and AST < 2.5 x ULN or < 5.0 x ULN with documented liver metastases - Total bilirubin < 1.5 x ULN   **SCOUT (LOXO-TRK-15003): *Laetsch et al. Lancet Oncol. 2018;19:705-14; DuBois et al. Cancer. 2018;124:4241-7.***[39, 40]  **Inclusion criteria**   - Bilirubin ≤ 1.5 x ULN for age - ALT ≤ 135 U/L   **NAVIGATE (LOXO-TRK-15002):** ***Drilon et al. N Engl J Med. 2018;378:731-9.***[41]  **Inclusion criteria**  Adequate hepatic function, as defined by:   - ALT or AST < 2.5 x ULN, or < 5 x ULN if due to underlying malignancy - Total bilirubin < 2.5 x ULN | Yes  Yes  Yes | **FDA; PI**  **Study details**   - Single oral 100-mg dose - No data on underlying cause of hepatic impairment   **Patients: Results**   - **Normal hepatic function** - **Child-Pugh class A:**   AUC_0-INF_ increased 1.3-fold;  C_max_ similar to subjects with  normal hepatic function   - **Child-Pugh class B:**   AUC_0-INF_ increased 2-fold;  C_max_ similar to subjects with  normal hepatic function   - **Child-Pugh class C:**   AUC_0-INF_ increased 3.2-fold;  C_max_ increased 1.5-fold  **EMA; SmPC**  **Study details**   - Single 100-mg dose - No data on underlying cause of hepatic impairment   **Patients: Results**   - **Normal hepatic function** - **Child-Pugh class A, B, and C:**   An increase in AUC_0-inf_ 1.3, 2 and 3.2-fold respectively. Cmax was increased by 1.1, 1.1 and 1.5-fold respectively. |

| Lenvatinib | **FDA; PI**   - **Child-Pugh class A**: No dose modification recommended - **Child-Pugh class B**: No dose modifications recommended for DTC, RCC, or EC; there is no recommended dose for patients with HCC - **Child-Pugh class C**: Reduce the dose to 14 mg once daily for patients with DTC, and 10 mg once daily for patients with RCC or EC; there is no recommended dose for patients with HCC   **EMA; SmPC**   - **Child-Pugh class A:** No starting dose adjustments are necessary for patients with DTC or HCC - **Child-Pugh class B**: No starting dose adjustments are necessary in patients with DTC; Insufficient data for dose recommendation in patients with HCC - **Child-Pugh class C**: Recommended starting dose is 14 mg once daily in patients with DTC; Not recommended for use in HCC | **SELECT: *Schlumberger et al. N Engl J Med. 2015;372:621-30.***[42]  **Inclusion criteria**  Adequate liver function, as defined by:   - Bilirubin ≤ 1.5 x ULN except for unconjugated hyperbilirubinemia or Gilbert’s syndrome - ALP, ALT, and AST ≤ 3 x ULN (≤ 5 x ULN if subject has liver metastases)     **Study 205: *Motzer et al., Lancet Oncol. 2015;16:1473-82.***[43]  **Inclusion criteria**   - Adequate hepatic function   **REFLECT: *Kudo et al. Lancet. 2018;391:1163-73.***[44]  **Inclusion criteria**   - Child-Pugh class A - Adequate liver function, as defined by: - Albumin ≥ 2.8 g/dL - Bilirubin ≤ 3.0 g/dL - AST, ALP, and ALT ≤ 5 x ULN | Yes  No  No | ***Shumaker et al. J Clin Pharmacol 2015;55:317–27.***[45]  **Study details**   - Single oral 10-mg dose (5-mg dose in Child-Pugh class C) - Subjects with acute liver disease, acute liver injury, or laboratory signs of acute, active HAV, HBV, or HBC were excluded (subjects with stable, chronic, active HBC or HCV permitted at investigator’s discretion)   **Patients: Results**   - **Normal hepatic function (n = 8)** - **Child-Pugh class A (n = 6):**   Total lenvatinib AUC_0-inf_ GMR: 1.2 (90% CI 0.8–1.8)   - **Child-Pugh class B (n = 6):**   Total lenvatinib AUC_0-inf_ GMR: 1.1 (90% CI 0.7–1.6)   - **Child-Pugh class C (n = 6):**   Total lenvatinib AUC_0-inf_ GMR: 1.8 (90% CI 1.2–2.7)  ***Ikeda et al. Clin Cancer Res. 2016;22:1385-94.***[46]  **Study details**   - Dose-escalation and PK study with 8-mg, 12-mg, 16-mg dose levels in advanced HCC - Patients with advanced HCC, stratified by hepatic function - Cause of HCC was HBV, HCV, alcohol, or unknown   **Patients: Results**   - **Solid tumors (n=25)** - **Child-Pugh class A (n = 9):**   MTD of lenvatinib was 12-mg once daily   - C_max_ of lenvatinib at 12-mg dose was 212 ng/mL (similar to that in patients with solid tumors [260 ng/mL]) - **Child-Pugh class B (n = 11):**   MTD of lenvatinib was 8-mg once daily   - No conclusive PK data for the Child-Pugh class B cohort; this is due to an insufficient number of patients (n=2) receiving 12-mg dose for comparison to solid tumor controls   ***Tamai et al. J Clin Pharmacol. 2017;57:1138-47.***[47]  **Study details**   - Pooled data analysis based on 15 studies and population PK modeling - Healthy subjects (n=232) - Subjects with HCC (n= 65) - Subject with other tumors (n=155) - Cause of HCC was HBV, HCV, or other   **Patients: Results**   - **Child-Pugh classes A and B:**   No differences in CL/F were observed between these two groups   - 12-mg (patients ≥ 60 kg) and 8-mg (patients < 60 kg) starting doses are recommended for patients with HCC Child-Pugh class A |
| --- | --- | --- | --- | --- |
| Lorlatinib | **FDA; PI**   - **Mild hepatic impairment (total bilirubin ≤ ULN and AST > ULN, or total bilirubin > 1 to 1**.**5 x ULN and any AST):** No dose adjustment required - **Moderate or severe hepatic impairment (definition not specified):** A recommended dose has not been established   **EMA; SmPC**   - **Mild hepatic impairment (definition not specified)^a^:** Population PK studies showed no clinically meaningfully effect on lorlatinib exposure; No dose adjustment recommended - **Moderate or severe hepatic impairment (definition not specified)^b^**: No data available; not recommended for use in these patients   ***^a^Criteria not defined***  ***^b^Clinical studies that were conducted excluded patients with AST or ALT > 2.5 × ULN, or if due to underlying malignancy, > 5.0 × ULN or with total bilirubin > 1.5 × ULN*** | **Study B7461001: *Solomon et al. Lancet Oncol. 2018;19:1654-67.***[48]  **Inclusion criteria**  Adequate liver function, as defined by:   - Total serum bilirubin ≤ 1.5 x ULN - AST and ALT ≤ 2.5 x ULN (≤ 5.0 x ULN in the event of liver metastases)   **Exclusion criteria**   - Active and clinically significant viral infection including HBV or HCV or known HIV | Yes | **N/A** |
| Midostaurin | **FDA; PI**   - **Mild (total bilirubin > 1–1.5 x ULN or AST > ULN) or moderate (total bilirubin 1.5–3.0 x the ULN and any AST) hepatic impairment:** did not have clinically meaningful effects on PK; No dosing guidance provided for patients with underlying hepatic impairment **Severe hepatic impairment (bilirubin ≥ 3 x ULN and any value for AST):** PK unknown; no dosing guidance provided   **EMA; SmPC**   - **Child-Pugh classes A and B**: No dose adjustments necessary - **Child-Pugh class C**: No study has been completed in patients with severe hepatic impairment | **CALGB 10603: *Stone et al. N Engl J Med. 2017;377:454-64.***[49]  **Inclusion criteria**   - Bilirubin < 2.5 x ULN   **CPKC412D2201: *Gotlib et al. N Engl J Med. 2016;374:2530-41.***[50]  **Inclusion criteria**   - AST and ALT ≤ 2.5 x ULN (≤ 5.0 x ULN if the elevation is solely due to ASM/MCL) - Serum bilirubin ≤ 1.5 x ULN (≤ 3.0 x ULN if the elevation is solely due to ASM/MCL)   **Exclusion criteria:**   - Known HIV or active viral hepatitis | Yes  Yes | **EMA; SmPC**  **Study details**   - 50-mg doses twice daily for 6 days - No data on underlying cause of hepatic impairment   **Patients: Results**   - **Healthy subjects** - **Child-Pugh class A:**   AUC_tau_ was decreased by 28% on day 7   - **Child-Pugh class B:**   AUC_tau_ was decreased by 20% on day 7   - **Child-Pugh class C:**   Not studied |
| Neratinib | **FDA; PI**   - **Child-Pugh classes A and B**: No dose adjustments are necessary - **Child-Pugh class C**: Reduce starting dose to 80 mg   **EMA; SmPC**   - **Child-Pugh classes A and B**: No dose adjustments necessary - **Child-Pugh class C**: Contraindicated | **ExteNET trial:** ***Chan et al. Lancet Oncol. 2016;17:367-77.***[51]  **Inclusion criteria**   - Normal organ function | No | **FDA; PI**  **Study details**   - Single 120-mg dose - Participants with chronic liver disease not due to cancer   **Patients: Results**   - **Healthy subjects (n = 9)** - **Child-Pugh class A (n = 6):** - Similar exposure to healthy subjects - **Child-Pugh class B (n = 6):**  Similar exposure to healthy subjects - **Child-Pugh class C (n = 6):**   C_max_ increased by 173%;  AUC increased by 181%  **EMA; SmPC**  **Study details**   - Dose not reported - Subjects without cancer   **Patients: Results**   - **Healthy subjects** - **Child-Pugh Class C:**   Clearance of neratinib decreased by 36% and exposure increased by ~3-fold compared to subjects with normal hepatic function |
| Niraparib | **FDA; PI**   - **Mild hepatic impairment^a^**: No dose adjustment required - **Moderate to severe hepatic impairment^a^**: Safety unknown   ***^a^NCI-ODWG criteria***  **EMA; SmPC guidance**   - **Mild-to-moderate hepatic impairment^b^**: No dose adjustment required - **Severe hepatic impairment^b^**: No data   ***^b^Criteria not defined*** | **ENGOT-OV16 / NOVA: *Mirza et al. N Engl J Med. 2016;375:2154-64.***[52]  **Inclusion criteria**  Adequate organ function, as defined by:   - Total bilirubin ≤ 1.5 ULN, or direct bilirubin ≤ 1.0 x ULN - AST and ALT ≤ 2.5 x ULN (≤ 5.0 x ULN if liver metastases are present)   **Exclusion criteria**   - Known active hepatic disease | Yes | **N/A** |
| Olaparib | **FDA; PI**   - **Child-Pugh classes A and B:** No dose adjustment needed - **Child-Pugh class C**: No data available   **EMA; SmPC**   - **Child-Pugh classes A and B**: No dose adjustment required - **Child-Pugh class C**: Not studied; Not recommended for use | ***Study 19: Ledermann et al. N Engl J Med. 2012;366:1382-92.***[53]  **Inclusion criteria**   - Total bilirubin ≤ 1.5 x institutional ULN - AST/ALT ≤ 2.5 x institutional ULN (≤ 5 x ULN if liver metastases are present)   **Exclusion criteria**   - Known hepatic disease (HBV or HCV)   **SOLO2 study:** ***Pujade-Lauraine et al. Lancet Oncol. 2017;18:1274–84.***[54]  **Inclusion criteria**   - Total bilirubin ≤ 1.5 x institutional ULN - AST/ALT ≤ 2.5 x institutional ULN (≤ 5 x ULN if liver metastases are present)   **Exclusion criteria**   - Known active hepatitis (HBV or HCV)   ***OlympiAD: Robson et al. N Engl J Med. 2017;377:523-33.***[55]  **Inclusion criteria**   - Total bilirubin ≤ 1.5 x institutional ULN - AST/ALT ≤ 2.5 x institutional ULN (unless liver metastases are present in which case, they must be ≤ 5.0 x ULN) | Yes  Yes  Yes | ***Pilla Reddy et al. Clin Pharmacol Ther. 2019;105:229-41.***[56]  ***Rolfo et al. Br J Clin Pharmacol. 2020;86:1807-18.****[57]*  **Study details**   - Single oral 300-mg dose - Patients with advanced solid tumors with normal, mild or moderate hepatic function (defined as: normal, AST or ALT ≤ 2.5 x institutional ULN unless liver metastases are present in which case, it must be ≤ 5.0 x ULN; mild, Child-Pugh class A; moderate, Child-Pugh class B). Patients with severe hepatic impairment were excluded.   **Patients: Results**   - **Normal hepatic function (n = 13)** - **Child-Pugh class A (n = 9):**   AUC GMR: 1.15 (90% CI: 0.72–1.83); C_max_ GMR: 1.13 (90% CI: 0.82–1.56)   - **Child-Pugh class B (n = 8):**   AUC GMR: 1.08 (90% CI: 0.66–1.74); C_max_ GMR: 0.87 (90% CI: 0.63, 1.22) |
| Osimertinib | **FDA; PI**   - **Child-Pugh classes A and B**: No dose adjustments necessary - **Mild to moderate hepatic impairment**   **(total** **bilirubin** ≤ **ULN and AST > ULN, or total bilirubin > 1 to 3 x ULN and any AST):** No dose adjustments necessary   - **Severe hepatic impairment (total bilirubin between 3 to 10 x ULN and any AST):** No recommended dose has been established   **EMA; SmPC**   - **Child-Pugh classes A and B**: No dose adjustments necessary - **Mild hepatic impairment (total** **bilirubin** ≤ **ULN and AST > ULN, or total bilirubin > 1.0 to 1.5 x ULN and any AST):** No dose adjustments necessary - **Moderate hepatic impairment (total bilirubin between 1.5 to 3 x ULN and any AST):** No dose adjustments necessary - **Severe hepatic impairment (definition not specified):** Safety unknown; use is not recommended | **AURA1: *Jänne et al. N Engl J Med. 2015;372:1689-99.***[58]  **Exclusion criteria:**   - Active infection, including HBV, HCV, and HIV - Inadequate hepatic function, as defined by: - AST and ALT > 2.5 x ULN (> 5 x ULN if liver metastases are present) - Total bilirubin > 1.5 x ULN (> 3 x ULN in the presence of liver metastases or Gilbert’s syndrome)   **AURA2: *Goss et al. Lancet Oncol. 2016;17:1643-52.***[59]  **Exclusion criteria:**   - Active infection, including HBV, HCV, and HIV - Inadequate hepatic function, as defined by: - AST and ALT > 2.5 x ULN (> 5 x ULN if liver metastases are present) - Total bilirubin > 1.5 x ULN (> 3 x ULN in the presence of liver metastases or Gilbert’s syndrome)   **AURA3: *Mok et al. N Engl J Med. 2017;376:629-40.***[60]  **Exclusion criteria:**   - Active infection, including HBV, HCV, and HIV - Inadequate hepatic function, as defined by: - AST and ALT > 2.5 x ULN (> 5 x ULN if liver metastases are present) - Total bilirubin > 1.5 x ULN (> 3 x ULN in the presence of liver metastases or Gilbert’s syndrome)   **FLAURA: *Soria et al. N Engl J Med. 2018;378:113-25.***[61]  **Exclusion criteria**   - Active infection, including HBV, HCV, and HIV - Inadequate hepatic function, as defined by: - AST and ALT > 2.5 x ULN (> 5 x ULN if liver metastases are present) - Total bilirubin > 1.5 x ULN (> 3 x ULN in the presence of liver metastases or Gilbert’s syndrome)   **AURA Extension: *Yang et al. J Clin Oncol. 2017;35:1288-96.***[62]  **Exclusion criteria**   - Active infection, including HBV, HCV, and HIV - Inadequate hepatic function, as defined by: - AST and ALT > 2.5 x ULN (> 5 x ULN if liver metastases are present) - Total bilirubin > 1.5 x ULN (> 3 x ULN in the presence of liver metastases or Gilbert’s syndrome) | Yes  Yes  Yes  Yes  Yes | ***Grande et al. J Pharmacol Exp Ther. 2019;369:291-99.***[63]  **Study details**   - Single 80-mg oral dose - Subjects with malignant solid tumors   **Patients: Results**   - **Normal hepatic function (n = 10)** - **Child-Pugh class A (n = 7):**   AUC GMR reduced to 63% (90% CI: 47.3-84.5); C_max_ reduced to 51% (90% CI: 36.6–72.3)   - **Child-Pugh class B (n = 5):**   AUC reduced to 68% (90% CI: 49.6–94.2); C_max_ reduced to 61% (90% CI: 41.6–88.6) |
| Palbociclib | **FDA; PI/EMA; SmPC**   - **Child-Pugh classes A and B**: No dose adjustments required - **Child-Pugh class C**: Recommended dose is 75 mg once daily for 21 consecutive days, followed by 7 days off-treatment (to comprise a complete 28-day cycle) | **PALOMA-2: *Finn et al. N Engl J Med. 2016;375:1925-36.***[64]  **Inclusion criteria**   - Total serum bilirubin ≤ 1.5 x ULN (≤ 3.0 ULN if Gilbert’s disease); - AST and/or ALT ≤ 3 x ULN (≤ 5 x ULN if liver metastases present) - ALP ≤ 2.5 x ULN (≤ 5 x ULN if bone or liver metastases present)   **PALOMA-3: *Turner et al. N Engl J Med 2015;373:209-19.***[65]  **Inclusion criteria**   - Total serum bilirubin ≤ 1.5 x ULN (< 3.0 ULN if Gilbert’s disease) - AST and/or ALT ≤ 3 x ULN (≤ 5 x ULN if liver metastases present) - ALP ≤ 2.5 x ULN (≤ 5 x ULN if bone or liver metastases present) | Yes  Yes | **FDA; PI and EMA; SmPC**  **Study details**   - Dose not reported - No data on underlying cause of hepatic impairment   **Patients: Results**   - **Normal hepatic function** - **Child-Pugh class A:**   Unbound AUC_inf_ decreased by 17%;  unbound C_max_ increased by 7%   - **Child-Pugh class B:**   Unbound AUC_inf_ increased by 34% unbound C_max_ increased by 38%   - **Child-Pugh class C**:   Unbound AUC_inf_ increased by 77% unbound C_max_ increased by 72% |
| Panobinostat | **FDA; PI**   - **Mild hepatic impairment (bilirubin ≤1 x ULN and AST > 1 x ULN, or bilirubin > 1 to 1.5 x ULN and any AST)^a^**: Reduce starting dose to 15 mg - **Moderate hepatic impairment (bilirubin > 1.5x to 3 x ULN, any AST)^a^**: Reduce starting dose to 10 mg - **Severe hepatic impairment**: Avoid use   ***^a^Definition not specified in ‘Dose Modifications for Use in Hepatic Impairment’, but inferred from information within the PI, because  NCI-ODWG criteria is referenced***  **EMA; SmPC**   - **Mild hepatic impairment^b^ (bilirubin ≤ 1.0 x ULN and AST > ULN, or bilirubin > 1.0 to 1.5 x ULN and any AST):** Reduce starting dose to 15 mg during the first treatment cycle. A dose escalation from 15 mg to 20 mg may be considered based on patient tolerability - **Moderate hepatic impairment^b^ (bilirubin > 1.5 to 3.0 x ULN and any AST)**: Reduce starting dose to 10 mg during the first treatment cycle. A dose escalation from 10 mg to 15 mg may be considered based on patient tolerability - **Severe hepatic impairment^b^:** Safety unknown; should not be administered     ^b^Based on NCI-CTEP classification | **PANORAMA 1: *San-Miguel et al. Lancet Oncol. 2014; 15:1195-206.***[66]  **Inclusion criteria**   - Normal liver function (defined by each institution)   **Study DUS71 – PANORAMA 2: *Richardson et al. Blood. 2013;122:2331-7.***[67]  **Inclusion criteria**   - AST and ALT ≤ 2.5 x ULN - Serum total bilirubin ≤ 1.5 x ULN (≤ 3.0 x ULN if Gilbert’s syndrome is present) | No  No | **EMA; SmPC**  ***Slingerland et al. Cancer Chemother Pharmacol. 2014;74:1089-1098.***[68]  **Study details**   - Single oral 30-mg dose - Subjects with advanced solid tumors (liver metastases and biliary stents permitted; use of medication that affects hepatic function not permitted)   **Patients: Results**   - **Normal hepatic function (n = 10)** - **Child-Pugh class A (n = 7)**   Median panobinostat exposure increased by 51%  Systemic exposure (measured by geometric mean of AUC_0-inf_) increased by 43% (1.4-fold) compared to normal controls   - **Child-Pugh class B (n = 7)**   Median panobinostat exposure increased by 56%  Systemic exposure (measured by geometric mean of AUC_0-inf_) increased by 105% (2-fold) compared to normal controls |
| Ribociclib | **FDA; PI/EMA; SmPC**   - **Child-Pugh class A**: No dose adjustments necessary - **Child-Pugh classes B and C**: Recommended starting dose is  400 mg once daily | **MONALEESA-2** (**CLEE011A2301): *Hortobagyi et al. N Engl J Med. 2016;375:1738-48.***[69]  **Inclusion criteria**  Adequate hepatic function, as defined by:   - AST and ALT < 2.5 x ULN (< 5 x ULN if liver metastases are present) - Total serum bilirubin < ULN (total bilirubin ≤ 3.0 x ULN with direct bilirubin within normal range in patients with Gilbert’s syndrome)   **Exclusion criteria**   - Known history of HIV infection or chronic active hepatitis   **MONALEESA-7 (CLEE011E2301): *Tripathy et al. Lancet Oncol. 2018;19:904-15.***[70]  **Inclusion criteria**  Adequate hepatic function, as defined by:   - AST and ALT < 2.5 x ULN (< 5 x ULN if liver metastases are present) - Total serum bilirubin < ULN (total bilirubin ≤ 3.0 x ULN with direct bilirubin < 1.5 x ULN in patients with Gilbert’s syndrome)   **Exclusion criteria**   - Known history of HIV infection or chronic active hepatitis   **MONALEESA-3 (CLEE011F2301): *Slamon et al. J Clin Oncol. 2018;36:2465-72.***[71]  **Inclusion criteria**  Adequate hepatic function, as defined by:   - AST and ALT < 2.5 x ULN (< 5 x ULN if liver metastases are present) - Total bilirubin < ULN (total bilirubin ≤ 3.0 x ULN, or direct bilirubin ≤ 1.5 x ULN in patients with Gilbert’s syndrome)   **Exclusion criteria**   - Child-Pugh class B or C - Known history of HIV infection or chronic active hepatitis | Yes  Yes  Yes | **FDA; PI**  **Study details**   - Dose not reported - Subjects with hepatic impairment   **Patients: Results**   - **Child-Pugh class A:**   No effect on exposure   - **Child-Pugh class B:**   AUC_inf_ GMR: 1.28; C_max_ GMR: 1.44   - **Child-Pugh class C:**   AUC_inf_ GMR: 1.29; C_max_ GMR: 1.32 |
| Rucaparib | **FDA; PI**   - **Mild hepatic impairment (total bilirubin ≤ ULN and AST > ULN, or total bilirubin 1.0–1.5 x ULN and any AST):** No dose adjustments are recommended - **Moderate to severe hepatic impairment (total bilirubin > 1.5 x ULN):** No data available; no recommendation   **EMA; SmPC**   - **Mild hepatic impairment (total bilirubin ≤ ULN and AST > ULN or total bilirubin > 1 to 1.5 x ULN and any AST)^a^:** No dose adjustments recommended - **Moderate or severe hepatic impairment (total bilirubin > 1**.**5 x ULN):** Limited data available; Not recommended for use   ***^a^Definition not specified in dosing section, but inferred from information within the SmPC, because*** *li****ver-function tests were referenced*** | **ARIEL2*: Swisher et al. Lancet Oncol. 2017;18:75-87.***[72]  **Inclusion criteria**  Adequate hepatic function with 14 days prior to the first dose, as defined by:   - AST and ALT ≤ 3 × ULN (≤ 5 × ULN if liver metastases are present) - Bilirubin ≤ 1.5 × ULN (< 2 x ULN if due to Gilbert’s syndrome)   **Exclusion criteria**   - Known HIV or history of chronic HBV or HCV   **ARIEL3*: Coleman et al. Lancet. 2017;390:1949-61.***[73]  **Inclusion criteria**  Adequate hepatic function within 14 days prior to the first dose, as defined by:   - AST and ALT ≤ 3 × ULN (≤ 5 x ULN if liver metastases are present) - Bilirubin ≤ 1.5 x ULN (< 2 x ULN if due to Gilbert’s syndrome)   **Exclusion criteria**   - Known HIV or history of chronic HBV or HCV   **Study 10: *Kristeleit et al. Clin Cancer Res. 2017;23:4095-106.***[74]  **Inclusion criteria**   - Adequate hepatic function | Yes  Yes  No | **N/A** |
| Sonidegib | **FDA; PI**   - **Child-Pugh class A, B, and C:** No dosing guidance provided because there was no clinically meaningful effect on exposure provided for patients with underlying hepatic impairment   **EMA; SmPC**   - **Hepatic impairment (Child-Pugh class A, B, and C):** No dose adjustment needed | **BOLT trial: *Migden et al. Lancet Oncol. 2015;16:716-28.***[75]  **Inclusion criteria**  Adequate hepatic function, as defined by:   - Total serum bilirubin ≤ 1.5 x ULN - AST and ALT ≤ 2.5 x ULN (≤ 5.0 x ULN if liver metastases are present) | No | ***Horsmans et al. Clin Pharmacokinet. 2018;57:345-54.***[76]  **Study details**   - Single oral 800-mg dose - Subjects with hepatic impairment had to have histologically or clinically confirmed cirrhosis   **Patients: Results**   - **Normal hepatic function (n = 8)** - **Child-Pugh class A (n = 8):**   C_max_ was 20% lower, C_max_ GMR: 0.80 (90% CI: 0.46–1.37); AUC_0-inf_ GMR: 0.60 (90% CI: 0.34–1.07)   - **Child-Pugh class B (n = 8):**   C_max_ was 21% lower, C_max_ GMR: 0.79 (90% CI: 0.46–1.36); AUC_0-inf_ 0.78 (90% CI 0.39–1.54)   - **Child-Pugh class C (n = 9):**   C_max_ was 60% lower, C_max_ GMR: 0.40 (90% CI: 0.24–0.68); AUC_0-inf_ GMR: 0.92 (90% CI: 0.46–1.82) |
| Talazoparib | **FDA; PI**   - **Mild hepatic impairment (total** **bilirubin ≤ 1 x ULN and AST > ULN, or total bilirubin > 1.0 to 1.5 x ULN and any AST):** No dose adjustments required - **Moderate hepatic impairment (total bilirubin > 1.5 to 3.0 x ULN and any AST) or severe hepatic impairment (total bilirubin > 3.0 x ULN and any AST):** Not studied; no guidance provided   **EMA; SmPC**   - **Mild hepatic impairment (total bilirubin ≤ ULN and AST > ULN or total bilirubin > 1**.**0 to 1**.**5 x ULN and any AST):** No dose adjustments required - **Moderate (total bilirubin > 1.5–3**.**0 x ULN and any AST) and severe (total bilirubin > 3**.**0 x ULN and any AST):** No data. Should be used only if benefit outweighs potential risk, and patient should be carefully monitored | **EMBRACA Study: *Litton et al. N Engl J Med. 2018;379:753-63.***[77]  **Inclusion criteria**  Adequate hepatic function, as defined by:   - Serum AST and ALT ≤ 2.5 x ULN (≤ 5 x ULN if due to hepatic metastasis) - Total serum bilirubin ≤ 1.5 x ULN (≤ 3 x ULN for Gilbert’s syndrome)   **Exclusion criteria**   - Known HIV or known active HBV or HCV | Yes | **N/A** |
| Trabectedin | **FDA; PI**   - **Mild hepatic impairment (bilirubin > 1 to 1.5 x ULN, and any AST or ALT):** No dose modification - **Moderate hepatic impairment (bilirubin > 1.5 to 3 x ULN, and AST and ALT < 8 x ULN):** Recommended dose is 0.9 mg/m^2^ - **Severe hepatic impairment (bilirubin > 3 ULN and any AST and ALT):** Contraindicated   **EMA; SmPC**  **The following criteria are required for treatment:**   - Bilirubin ≤ ULN - ALT and AST ≤ 2.5 x ULN - ALP ≤ 2.5 x ULN - Albumin ≥ 25 g/L | **ET743-SAR-3007:** ***Demetri et al. J Clin Oncol. 2009;27:4188-96.***[78]  **Inclusion criteria**   - Bilirubin ≤ ULN - AST/ALT ≤ 2.5 x ULN - Total ALP ≤ ULN (if total ALP > ULN, the AP liver fraction or 5ʹ-nucleotidase had to be ≤ ULN) - Albumin > 2.5 g/dL   **Exclusion criteria**   - Active viral hepatitis or chronic liver disease   **ET743-OVA-301: *Monk et al. J Clin Oncol. 2010;28:3107-14.***[79]  **Inclusion criteria**   - Total bilirubin ≤ 1.5 x ULN - Direct bilirubin ≤ ULN - Total ALP ≤ 1.5 x ULN (if total ALP > 1.5 x ULN, ALP liver fraction or 5ʹ-nucleotidase ≤ ULN) - ALT and AST ≤ 2.5 x ULN   **ET743-SAR- 3002: *Samuels et al. Ann Oncol. 2013; 24;1703-9.***[80]  **Inclusion criteria:**   - ALP, AST, ALT ≤ 2.5 x ULN   **Exclusion criteria**   - Active symptomatic viral hepatitis or chronic liver disease | No  No  No | **N/A** |
| Trifluridine and tipiracil hydrochloride | **FDA; PI**   - **Mild hepatic impairment (total bilirubin ≤ ULN and AST > ULN** - **or total bilirubin < 1 to 1.5 x ULN and any AST)^a^**: No dose modification required - **Moderate or severe hepatic impairment** **(total bilirubin > 1.5 x ULN and any AST)**: Do not administer   ***^a^Definition for mild hepatic impairment not specified in dosing, it was noted under ‘Specific Populations’***  **EMA; SmPC**   - **Mild hepatic impairment (NCI group B)^a^** No dose modification required - **Moderate or severe hepatic impairment** **(NCI criteria Groups C and D defined by total bilirubin > 1**.**5 x ULN):** Administration not recommended   ***^a^Definition for mild hepatic impairment not specified in dosing; it was noted in ‘Pharmacokinetics in Special Populations’*** | **RECOURSE trial: *Mayer et al. N Engl J Med. 2015;372:1909-19.***[81]  **Inclusion criteria**  Adequate hepatic function, as defined by:   - Total serum bilirubin of ≤ 1.5 mg/dL (except for grade 1 hyperbilirubinemia solely because of a medical diagnosis of Gilbert’s syndrome) - AST and ALT ≤ 3.0 x ULN (≤ 5.0 x ULN if due to underlying liver metastasis)   **Exclusion criteria**   - Known HIV, HBV, or HCV   **TAGS: *Shitara et al. Lancet Oncol. 2018;19:1437-48.***[82]  **Inclusion criteria**  Adequate hepatic function, as defined by:   - Total serum bilirubin ≤ 1.5 x ULN - AST and ALT ≤ 3.0 x ULN (≤ 5.0 x ULN if due to underlying liver metastasis) | Yes  Yes | **N/A** |
| Venetoclax | **FDA; PI**   - **Child-Pugh classes A and B:** No dose adjustments necessary - **Severe hepatic impairment (Child-Pugh class C):** Reduce once daily dose by 50%; monitor closely for signs of toxicity   **EMA; SmPC**   - **Mild and moderate hepatic impairment (Child-Pugh class A; total bilirubin and AST > ULN or total bilirubin >1.0 to 1.5 x ULN)^a^**: No dose adjustments are recommended - **Moderate hepatic impairment**   **(Child-Pugh Class B; bilirubin >1.5 to 3.0 x ULN,)^a^:** No dose adjustments are recommended; patients should be monitored closely for signs of toxicity   - **Severe hepatic impairment (Child-Pugh Class C; total bilirubin >3.0 x ULN)^a^:** A recommended dose reduction of at least 50% throughout the study; patients should be monitored closely for signs of toxicity   ***^a^Definition not given within dosing; it was noted within the SmPC under ‘Special Populations’.*** | **MURANO study:** ***Seymour et al. N Engl J Med. 2018;378:1107-20.***[83]  **Inclusion criteria**  Adequate hepatic function at screening, as defined by:   - AST and ALT ≤ 3.0 x ULN - Bilirubin ≤ 1.5 x ULN (> 1.5 x ULN may be permitted if due to Gilbert’s syndrome)   **Exclusion criteria**   - Known HIV or positive hepatitis serology   **Study M12-175: *Roberts et al. N Engl J Med. 2016;374:311-22.***[84]  **Inclusion criteria**  Adequate hepatic function at screening, as defined by:   - AST and ALT ≤ 3.0 x ULN - Bilirubin ≤ 1.5 x ULN (> 1.5 x ULN may be permitted if due to Gilbert’s syndrome)   **Exclusion criteria**   - Known HIV or significant history of hepatic disease   **Study M13-982: *Stilgenbauer et al. Lancet Oncol. 2016;17:768-78.***[85]  **Inclusion criteria**  Adequate hepatic function at screening, as defined by:   - AST and ALT ≤ 3.0 × ULN - Bilirubin ≤ 1.5 × ULN. Subjects with Gilbert's Syndrome may have a bilirubin > 1.5 × ULN, per discussion between the investigator and AbbVie medical monitor   **Exclusion criteria**   - Subject has a significant history of hepatic disease that in the opinion of the investigator would adversely affect his/her participating in this study   ***Study M14-032: Jones et al. Lancet Oncol. 2018;19:65-75.***[86]  **Inclusion criteria**  Adequate hepatic function at screening, as defined by:   - AST and ALT ≤ 1.5 x ULN - Bilirubin ≤ 1.5 x ULN. Subjects with AIHA and Gilbert's Syndrome may have a bilirubin > 1.5 × ULN, per discussion between the investigator and AbbVie medical monitor   **Exclusion criteria**   - Known HIV, or chronic HBV or HCV infection requiring treatment   **Study M14-358: *DiNardo et al. Blood. 2019; 133:7-17.***[87]  **Inclusion criteria**  Adequate hepatic function, as defined by:   - AST and ALT ≤ 2.5 x ULN (unless considered due to leukemic organ involvement) - Bilirubin ≤ 1.5 x ULN (unless considered due to leukemic organ involvement) Patients with Gilbert’s syndrome and bilirubin >1.5 x ULN may be permitted, at AbbVie medical monitor and investigator’s discretion   **Exclusion criteria**   - Known HIV or significant history of hepatic disease   **M14-387: *Wei et al. J Clin Oncol. 2019;20;37:1277-84.***[88]  **Inclusion criteria**  Adequate hepatic function  **Exclusion criteria**   - Known HIV | Yes  Yes  Yes  Yes  Yes  Yes | **EMA; SmPC**  **Study details**   - Single 50-mg dose - Dedicated hepatic impairment study   **Patients: Results**   - **Normal hepatic function (n = 6)** - **Child-Pugh class A (n = 6):**   C_max_ and AUC were similar to subjects with normal hepatic function after receiving a  single 50-mg dose   - **Child-Pugh class B (n = 6):** C_max_ and AUC were similar to subjects with normal hepatic function after receiving a  single 50-mg dose - **Child-Pugh class C (n = 5):** C_max_ similar to subjects with normal hepatic function; AUC_inf_ was on average 2.7-fold higher than AUC_inf_ in subjects with normal hepatic function |

This list excludes monoclonal antibodies, autologous T cell immunotherapies, radionuclides, viruses, enzymes, and supportive care medications; drugs with an approval for an indication prior to 2014 were also excluded. Of note, cabozantinib was approved in 2012 as a capsule, however, it was included in this review because its first approval as a tablet was in 2016. Inclusion criteria is not an exhaustive list of all inclusion criteria for the clinical trials; instead it is a list of inclusion criteria relevant to hepatic function (eg, bilirubin, AST, and ALT levels). The exclusion criteria listed is not a complete list of all exclusion criteria but instead aims to capture parameters associated with liver impairment (eg, HBV, HCV, HIV, cirrhosis). This table is based on prescribing information and/or summary of product characteristics that were available for each drug as of August 2020.

ASM, aggressive systemic mastocytosis; AST, aspartate aminotransferase; ALP, alkaline phosphatase; ALT, alanine aminotransferase; AUC, area under the curve; C_max_, maximum serum concentration; EMA, European Medicines Agency; FDA, US Food and Drug Administration; GMR, geometric mean ratio; HAV, hepatitis A; HBsAg, Australia antigen, surface antigen of the hepatitis B virus; HBV, hepatitis B virus; HCV, hepatitis C virus; HCC, hepatocellular carcinoma; HIV, human immunodeficiency virus; MCL, mast cell leukemia; NCI-ODWG, National Cancer Institute Organ Dysfunction Working Group; OD, once daily; PI, prescribing information; PK, pharmacokinetic; RCC, renal cell carcinoma; RNA, ribonucleic acid; SGOT, serum glutamic-oxaloacetic transaminase; SGPT, serum glutamic pyruvic transaminase; SmPC, Summary of Product Characteristics; ULN, upper limit of normal.

**Supplemental Table 3.** Clinicaltrials.gov search results for the 39 drugs included in this narrative review: Criteria for search drug name *plus* Child-Pugh *OR* liver dysfunction *OR* hepatic impairment

| **Drug** | **Clinical trial**  **identifier** | **Study title** | **Publication**  ***Additional study details*** | **Study population** | **Criteria used for defining liver impairment** |
| --- | --- | --- | --- | --- | --- |
| Abemaciclib | NCT02387814 | A Study of Abemaciclib in Participants With Varying Degrees of Liver Impairment | No publication^a^ | Noncancer | Classified as mild, moderate, or severe *(criteria used was not defined)* |
| Acalabrutinib | NCT03968848 | Investigate the Influence of Severe Hepatic Impairment on the Pharmacokinetics of Acalabrutinib and Its Metabolite | Edlund et al. *Clin Pharmacokinet.* 2019 May;58(5):659-672.[89]^b^ | Normal hepatic function or severe hepatic impairment | Child-Pugh |
| Alectinib | NCT02621047 | Effect of Hepatic Impairment on the Pharmacokinetics of Alectinib | Morcos et al. *J Clin Pharmacol.* 2018; 58: 1618-1628.[8] | Normal hepatic function or moderate or severe hepatic impairment | Child-Pugh |
| Apalutamide | NCT04154774 | A Study of Apalutamide in Participants With Severe Hepatic Impairment Compared With Participants With Normal Hepatic Function | No publication  *Estimated Study Completion Date: March 15, 2021* | Normal hepatic function or severe hepatic impairment | Child-Pugh |
| Belinostat | NCT01273155 | Belinostat for Solid Tumors and Lymphomas in Patients With Varying Degrees of Hepatic Dysfunction | No publication^a^ | Solid tumors or lymphomas; normal hepatic function or mild, moderate, or severe hepatic impairment | Defined by the following liver function tests:   - Bilirubin - AST |
| Binimetinib | NCT02050815 | MEK162 in Healthy Subjects With Normal Hepatic Function and Subjects With Impaired Hepatic Function (MEK162) | No publication  *Study terminated; the Sponsor is terminating the study because the primary objective was achieved after 5 of the 6 subjects were assessed in the final cohort* | Normal or impaired hepatic function | Impaired hepatic function defined by elevation in following liver function tests above ULN:   - Bilirubin - AST |
| Brigatinib | None | NA | NA | NA | NA |
| Cabozantinib | NCT01493869 | Study to Assess the Pharmacokinetics of Cabozantinib (XL184) in Hepatic Impaired Adult Subjects | No publication | Normal hepatic function or mild, moderate, or severe hepatic impairment | Child-Pugh |
| Cabozantinib | NCT04454762 | A Study to Evaluate the Safety, Tolerability and Efficacy of Cabozantinib in Patients With Hepatocellular Carcinoma and Impaired Liver Function (CaboCHILD) | No publication  *Study is recruiting* | Hepatocellular carcinoma; impaired liver function | Impaired liver function defined as Child-Pugh class B |
| Ceritinib | NCT01950481 | Effect of Hepatic Impairment on LDK378 Pharmacokinetics | Publication not found  *Study completed September 2016* | Normal hepatic function or mild, moderate, or severe hepatic impairment | Criteria used was not defined |
| Cobimetinib | NCT02300025 | A Study to Evaluate the Pharmacokinetics and Safety of Cobimetinib in Volunteers With and Without Liver Damage | Publication not found  *Study completed January 2015* | Normal hepatic function or mild, moderate, or severe hepatic impairment | Child-Pugh |
| Copanlisib | NCT03172884 | Study of Copanlisib in Hepatic or Renal Impairment | Publication not found  *Study completed May 2020* | Noncancer^c^; normal hepatic function or moderate or severe hepatic impairment | Child-Pugh |
| Dacomitinib | NCT03865446 | Evaluate Severe Hepatic Impairment on Dacomitinib PK | Publication not found  *Study completed October 2019* | Normal hepatic function or severe hepatic impairment | Criteria used was not defined |
| Dacomitinib | NCT01571388 | A Study to Compare Pharmacokinetics of Dacomitinib (PF-00299804) Between Healthy Subjects and Subjects With Mild and Moderate Hepatic Impairment | Giri et al 2015; *Invest New Drugs.* 2015 Aug;33(4):931-41.[24] | Normal hepatic function or mild or moderate hepatic impairment | Child-Pugh |
| Daunorubicin and cytarabine encapsulated in liposomes | None | NA | NA | NA | NA |
| Duvelisib | NCT02095587 | Duvelisib in Hepatically Impaired Subjects Compared to Healthy Subjects (IPI-145-14) | Publication not found  *Study completed October 2014* | Normal hepatic function or mild, moderate, or severe hepatic impairment | Criteria used was not defined |
| Enasidenib | NCT03290443 | A Study to Assess the Pharmacokinetics of Enasidenib (CC-90007) in Subjects With Moderate and Severe Hepatic Impairment | Publication not found  *Study completed October 2018* | Normal hepatic function or moderate or severe hepatic impairment | Child-Pugh |
| Enasidenib | NCT04573582 | Pharmacokinetics of Enasidenib (CC-90007) in Participants With Mild, Moderate and Severe Hepatic Impairment | Study is not yet recruiting | Normal hepatic function or mild, moderate, or severe hepatic impairment | Child-Pugh |
| Encorafenib | None | NA | NA | NA | NA |
| Gilteritinib | NCT02571816 | A Study to Investigate the Effect of Hepatic Impairment on the Pharmacokinetics, Safety and Tolerability of ASP2215 | James AJ et al. *Clin Pharmacokinet.* 2020 Oct;59(10):1273-1290.[90] | Noncancer; normal hepatic function or mild or moderate hepatic impairment | Child-Pugh |
| Glasdegib | NCT03627754 | A Study to Evaluate the Effect of Hepatic Impairment on the Pharmacokinetics of Glasdegib | Masters JC et al. *Clin Pharmacol* *Drug Dev*. 2020 Dec 23.[91] | Normal hepatic function or moderate or severe hepatic impairment | Child-Pugh |
| Idelalisib | None | NA | NA | NA | NA |
| Irinotecan in a pegylated liposomal formulation | None | NA | NA | NA | NA |
| Ivosidenib | NCT03282513 | A Study of AG-120 (Ivosidenib) in Subjects With Mild or Moderate Hepatic Impairment or Normal Hepatic Function | Fan B et al. *Clin Pharmacol Drug Dev*. 2021 Jan;10(1):99-109.[92] | Noncancer; normal hepatic function or mild or moderate hepatic impairment | Child-Pugh |
| Ixazomib | NCT01912222 | Pharmacokinetic Study of Oral IXAZOMIB in Cancer Patients With Liver Dysfunction | Gupta N et al. *Br J Clin Pharmacol.* 2016 Sep; 82(3): 728–738.[93] | Advanced solid tumors or hematologic malignancies; normal hepatic function or moderate or severe hepatic impairment | Defined by the following liver function tests:   - Bilirubin - AST |
| Larotrectinib | None | NA | NA | NA | NA |
| Lenvatinib | NCT02421042 | A Pharmacokinetic and Safety Study of E7080 in Subjects With Mild (10 mg), Moderate (10 mg), and Severe Hepatic Impairment (5 mg) and Normal Hepatic Function (10 mg) | Shumaker et al. *J Clin Pharmacol.* 2015 Mar;55(3):317-27.[45] | Normal hepatic function or mild, moderate, or severe hepatic impairment | Child-Pugh |
| Lenvatinib | NCT02998775 | Phase 0 Study in Healthy, Hepatic and Renal Impaired Subjects to Obtain Plasma for Lenvatinib Protein Binding | Publication not found  *Study completed July 2017* | Healthy, renal impairment (mild, moderate, severe), or hepatic impairment (mild, moderate, severe) | Child-Pugh |
| Lorlatinib | NCT03726333 | Hepatic Impairment Study for Lorlatinib in Cancer Patients | Study is recruiting | Cancer; mild, moderate, or severe hepatic impairment | Based on liver function *(exact criteria not defined)* |
| Midostaurin | NCT01429337 | PK and Safety of Midostaurin in Subjects With Impaired Hepatic Function and Subjects With Normal Hepatic Function | Publication not found  *Study completed May 2020* | Normal hepatic function or mild or moderate hepatic impairment | Child-Pugh |
| Neratinib | NCT00781430 | Study Evaluating The PK And Safety Of Neratinib In Healthy Subjects And Subjects With Chronic Liver Disease | Publication not found  *Study completed February 2010* | Chronic liver disease | Criteria used was not defined |
| Niraparib | NCT03359850 | Pharmacokinetic and Safety Study of Niraparib With Normal or Moderate Hepatic Impairment Patients | No publication^a^ | Advanced solid malignancy; normal or moderate hepatic impairment | Defined by the following liver function tests:   - Bilirubin - AST |
| Olaparib | NCT01894243 | Study to Assess the Blood Levels and Safety of Olaparib in Patients With Advanced Solid Tumours and Normal Liver Function or Mild or Moderate Liver Impairment | Rolfo C et al. *Br J Clin Pharmacol.* 2020 Sep;86(9):1807-1818.[57] | Advanced solid tumors; normal hepatic function or mild or moderate hepatic impairment | Child-Pugh |
| Osimertinib | NCT02161770 | Study to Assess the Blood Levels and Safety of AZD9291 in Patients With Advanced Solid Tumours and Normal Liver Function or Mild or Moderate Liver Impairment | Grande E et al. *J Pharmacol Exp Ther.* 2019 May;369(2):291-299.[63] | Advanced solid tumors; normal hepatic function or mild or moderate hepatic impairment | Child-Pugh |
| Palbociclib | NCT02334800 | A Study To Describe The Effect Of Impaired Hepatic Function Of The Pharmacokinetics Of Palbociclib | No publication^a^ | Noncancer | Child-Pugh |
| Panobinostat | NCT01007968 | Pharmacokinetics and Safety of Panobinostat in Patients With Advanced Solid Tumors and Various Degrees of Hepatic Function | Slingerland M et al. *Cancer Chemother Pharmacol.* 2014 Nov;74(5):1089-98.[68] | Advanced solid tumors; varying degrees of hepatic impairment | National Cancer Institute-Cancer Therapy Evaluation Program (NCI-CTEP) criteria  and Child-Pugh |
| Ribociclib | NCT02388620 | Evaluation of Hepatic Function Impairment on the Pharmacokinetics of LEE011 | Publication not found  *Study completed January 2017* | Varying degrees of hepatic impairment | Child-Pugh |
| Rucaparib | NCT03521037 | Rucaparib Hepatic Impairment Study in Patients With a Solid Tumor | Publication not found  *Study completion date: December 30, 2020* | Advanced solid tumor; normal hepatic function or moderate hepatic impairment | National Cancer Institute Organ Dysfunction Working Group (NCI-ODWG) criteria |
| Sonidegib | NCT01764776 | Effect of Hepatic Impairment on LDE225 | Publication not found  *Study completed March 2015* | Normal (healthy) or impaired hepatic function | Criteria used was not defined |
| Talazoparib | NCT02997176 | An Open-Label Pharmacokinetics and Safety Study of Talazoparib (MDV3800) | Publication not found  *Study completed February 2020* | Advanced solid tumor; normal hepatic function or mild, moderate, or severe hepatic impairment | National Cancer Institute Organ Dysfunction Working Group (NCI-ODWG) criteria |
| Trabectedin | NCT01273493 | A Pharmacokinetic Study of Trabectedin in Patients With Advanced Malignancies and Hepatic Dysfunction | Calvo et al. *Invest New Drugs.* 2018 Jun;36(3):476-486.[94] | Advanced malignancies; hepatic dysfunction | Defined by the following liver function tests:   - Bilirubin - AST - ALT - ALP |
| Trifluridine and tipiracil hydrochloride | None | NA | NA | NA | NA |
| Venetoclax | None | NA | NA | NA | NA |

^a^Unpublished pharmacokinetic data was available on clinicaltrials.gov.

^b^This was a population pharmacokinetic study and therefore was not included as peer-reviewed pharmacokinetic study in the main manuscript.

^c^Patients with curatively treated cervical cancer in situ, non-melanoma skin cancer, superficial bladder cancer as well as localized prostate cancer could enroll.

ALP; alkaline phosphatase; ALT; alanine aminotransferase; AST, aspartate aminotransferase; ULN, upper limit normal.

**Supplemental References**

1 Dickler MN, Tolaney SM, Rugo HS, et al. MONARCH 1, a phase II study of abemaciclib, a CDK4 and CDK6 inhibitor, as a single agent, in patients with refractory HR^+^/HER2^-^ metastatic breast cancer. *Clin Cancer Res* 2017;23:5218-24.

2 Sledge GW, Jr., Toi M, Neven P, et al. MONARCH 2: Abemaciclib in combination with fulvestrant in women with HR+/HER2- advanced breast cancer who had progressed while receiving endocrine therapy. *J Clin Oncol* 2017;35:2875-84.

3 Goetz MP, Toi M, Campone M, et al. MONARCH 3: Abemaciclib as initial therapy for advanced breast cancer. *J Clin Oncol* 2017;35:3638-46.

4 Wang M, Rule S, Zinzani PL, et al. Acalabrutinib in relapsed or refractory mantle cell lymphoma (ACE-LY-004): a single-arm, multicentre, phase 2 trial. *Lancet* 2018;391:659-67.

5 Ou SH, Ahn JS, De Petris L, et al. Alectinib in crizotinib-refractory ALK-rearranged non-small-cell lung cancer: a phase II global study. *J Clin Oncol* 2016;34:661-8.

6 Shaw AT, Gandhi L, Gadgeel S, et al. Alectinib in ALK-positive, crizotinib-resistant, non-small-cell lung cancer: a single-group, multicentre, phase 2 trial. *Lancet Oncol* 2016;17:234-42.

7 Peters S, Camidge DR, Shaw AT, et al. Alectinib versus crizotinib in untreated ALK-positive non-small-cell lung cancer. *N Engl J Med* 2017;377:829-38.

8 Morcos PN, Cleary Y, Sturm-Pellanda C, et al. Effect of hepatic impairment on the pharmacokinetics of alectinib. *J Clin Pharmacol* 2018;58:1618-28.

9 Smith MR, Saad F, Chowdhury S, et al. Apalutamide treatment and metastasis-free survival in prostate cancer. *N Engl J Med* 2018;378:1408-18.

10 O'Connor OA, Horwitz S, Masszi T, et al. Belinostat in patients with relapsed or refractory peripheral T-cell lymphoma: results of the pivotal phase II BELIEF (CLN-19) study. *J Clin Oncol* 2015;33:2492-9.

11 Dummer R, Ascierto PA, Gogas HJ, et al. Encorafenib plus binimetinib versus vemurafenib or encorafenib in patients with BRAF-mutant melanoma (COLUMBUS): a multicentre, open-label, randomised phase 3 trial. *Lancet Oncol* 2018;19:603-15.

12 Kim DW, Tiseo M, Ahn MJ, et al. Brigatinib in patients with crizotinib-refractory anaplastic lymphoma kinase-positive non-small-cell lung cancer: a randomized, multicenter phase II trial. *J Clin Oncol* 2017;35:2490-98.

13 Choueiri TK, Escudier B, Powles T, et al. Cabozantinib versus everolimus in advanced renal-cell carcinoma. *N Engl J Med* 2015;373:1814-23.

14 Choueiri TK, Halabi S, Sanford BL, et al. Cabozantinib versus sunitinib as initial targeted therapy for patients with metastatic renal cell carcinoma of poor or intermediate risk: the Alliance A031203 CABOSUN trial. *J Clin Oncol* 2017;35:591-97.

15 Abou-Alfa GK, Meyer T, Cheng AL, et al. Cabozantinib in patients with advanced and progressing hepatocellular carcinoma. *N Engl J Med* 2018;379:54-63.

16 Nguyen L, Holland J, Ramies D, et al. Effect of renal and hepatic impairment on the pharmacokinetics of cabozantinib. *J Clin Pharmacol* 2016;56:1130-40.

17 Shaw AT, Kim DW, Mehra R, et al. Ceritinib in ALK-rearranged non-small-cell lung cancer. *N Engl J Med* 2014;370:1189-97.

18 Soria JC, Tan DSW, Chiari R, et al. First-line ceritinib versus platinum-based chemotherapy in advanced ALK-rearranged non-small-cell lung cancer (ASCEND-4): a randomised, open-label, phase 3 study. *Lancet* 2017;389:917-29.

19 Shaw AT, Kim TM, Crinò L, et al. Ceritinib versus chemotherapy in patients with ALK-rearranged non-small-cell lung cancer previously given chemotherapy and crizotinib (ASCEND-5): a randomised, controlled, open-label, phase 3 trial. *Lancet Oncol* 2017;18:874-86.

20 Ribas A, Gonzalez R, Pavlick A, et al. Combination of vemurafenib and cobimetinib in patients with advanced BRAF^V600^-mutated melanoma: a phase 1b study. *Lancet Oncol* 2014;15:954-65.

21 Larkin J, Ascierto PA, Dréno B, et al. Combined vemurafenib and cobimetinib in BRAF-mutated melanoma. *N Engl J Med* 2014;371:1867-76.

22 Dreyling M, Morschhauser F, Bouabdallah K, et al. Phase II study of copanlisib, a PI3K inhibitor, in relapsed or refractory, indolent or aggressive lymphoma. *Ann Oncol* 2017;28:2169-78.

23 Wu YL, Cheng Y, Zhou X, et al. Dacomitinib versus gefitinib as first-line treatment for patients with EGFR-mutation-positive non-small-cell lung cancer (ARCHER 1050): a randomised, open-label, phase 3 trial. *Lancet Oncol* 2017;18:1454-66.

24 Giri N, Masters JC, Plotka A, et al. Investigation of the impact of hepatic impairment on the pharmacokinetics of dacomitinib. *Invest New Drugs* 2015;33:931-41.

25 Lancet JE, Uy GL, Cortes JE, et al. CPX-351 (cytarabine and daunorubicin) liposome for injection versus conventional cytarabine plus daunorubicin in older patients with newly diagnosed secondary acute myeloid leukemia. *J Clin Oncol* 2018;36:2684-92.

26 Flinn IW, Hillmen P, Montillo M, et al. The phase 3 DUO trial: duvelisib vs ofatumumab in relapsed and refractory CLL/SLL. *Blood* 2018;132:2446-55.

27 Flinn IW, Miller CB, Ardeshna KM, et al. DYNAMO: A phase II study of duvelisib (IPI-145) in patients with refractory indolent non-Hodgkin lymphoma. *J Clin Oncol* 2019;37:912-22.

28 Stein EM, DiNardo CD, Pollyea DA, et al. Enasidenib in mutant IDH2 relapsed or refractory acute myeloid leukemia. *Blood* 2017;130:722-31.

29 Perl AE, Martinelli G, Cortes JE, et al. Gilteritinib or chemotherapy for relapsed or refractory FLT3-mutated AML. *N Engl J Med* 2019;381:1728-40.

30 Cortes JE, Heidel FH, Hellmann A, et al. Randomized comparison of low dose cytarabine with or without glasdegib in patients with newly diagnosed acute myeloid leukemia or high-risk myelodysplastic syndrome. *Leukemia* 2019;33:379-89.

31 Gopal AK, Kahl BS, de Vos S, et al. PI3Kδ inhibition by idelalisib in patients with relapsed indolent lymphoma. *N Engl J Med* 2014;370:1008-18.

32 Furman RR, Sharman JP, Coutre SE, et al. Idelalisib and rituximab in relapsed chronic lymphocytic leukemia. *N Engl J Med* 2014;370:997-1007.

33 Jones JA, Robak T, Brown JR, et al. Efficacy and safety of idelalisib in combination with ofatumumab for previously treated chronic lymphocytic leukaemia: an open-label, randomised phase 3 trial. *Lancet Haematol* 2017;4:e114-26.

34 Jin F, Robeson M, Zhou H, et al. The pharmacokinetics and safety of idelalisib in subjects with moderate or severe hepatic impairment. *J Clin Pharmacol* 2015;55:944-52.

35 Wang-Gillam A, Li CP, Bodoky G, et al. Nanoliposomal irinotecan with fluorouracil and folinic acid in metastatic pancreatic cancer after previous gemcitabine-based therapy (NAPOLI-1): a global, randomised, open-label, phase 3 trial. *Lancet* 2016;387:545-57.

36 DiNardo CD, Stein EM, de Botton S, et al. Durable remissions with Ivosidenib in IDH1-mutated relapsed or refractory AML. *N Engl J Med* 2018;378:2386-98.

37 Moreau P, Masszi T, Grzasko N, et al. Oral ixazomib, lenalidomide, and dexamethasone for multiple myeloma. *N Engl J Med* 2016;374:1621-34.

38 Hong DS, Bauer TM, Lee JJ, et al. Larotrectinib in adult patients with solid tumours: a multi-centre, open-label, phase I dose-escalation study. *Ann Oncol* 2019;30:325-31.

39 Laetsch TW, DuBois SG, Mascarenhas L, et al. Larotrectinib for paediatric solid tumours harbouring NTRK gene fusions: phase 1 results from a multicentre, open-label, phase 1/2 study. *Lancet Oncol* 2018;19:705-14.

40 DuBois SG, Laetsch TW, Federman N, et al. The use of neoadjuvant larotrectinib in the management of children with locally advanced TRK fusion sarcomas. *Cancer* 2018;124:4241-47.

41 Drilon A, Laetsch TW, Kummar S, et al. Efficacy of larotrectinib in TRK fusion-positive cancers in adults and children. *N Engl J Med* 2018;378:731-39.

42 Schlumberger M, Tahara M, Wirth LJ, et al. Lenvatinib versus placebo in radioiodine-refractory thyroid cancer. *N Engl J Med* 2015;372:621-30.

43 Motzer RJ, Hutson TE, Glen H, et al. Lenvatinib, everolimus, and the combination in patients with metastatic renal cell carcinoma: a randomised, phase 2, open-label, multicentre trial. *Lancet Oncol* 2015;16:1473-82.

44 Kudo M, Finn RS, Qin S, et al. Lenvatinib versus sorafenib in first-line treatment of patients with unresectable hepatocellular carcinoma: a randomised phase 3 non-inferiority trial. *Lancet* 2018;391:1163-73.

45 Shumaker R, Aluri J, Fan J, et al. Influence of hepatic impairment on lenvatinib pharmacokinetics following single-dose oral administration. *J Clin Pharmacol* 2015;55:317-27.

46 Ikeda M, Okusaka T, Mitsunaga S, et al. Safety and pharmacokinetics of lenvatinib in patients with advanced hepatocellular carcinoma. *Clin Cancer Res* 2016;22:1385-94.

47 Tamai T, Hayato S, Hojo S, et al. Dose finding of lenvatinib in subjects with advanced hepatocellular carcinoma based on population pharmacokinetic and exposure-response analyses. *J Clin Pharmacol* 2017;57:1138-47.

48 Solomon BJ, Besse B, Bauer TM, et al. Lorlatinib in patients with ALK-positive non-small-cell lung cancer: results from a global phase 2 study. *Lancet Oncol* 2018;19:1654-67.

49 Stone RM, Mandrekar SJ, Sanford BL, et al. Midostaurin plus chemotherapy for acute myeloid leukemia with a FLT3 mutation. *N Engl J Med* 2017;377:454-64.

50 Gotlib J, Kluin-Nelemans HC, George TI, et al. Efficacy and safety of midostaurin in advanced systemic mastocytosis. *N Engl J Med* 2016;374:2530-41.

51 Chan A, Delaloge S, Holmes FA, et al. Neratinib after trastuzumab-based adjuvant therapy in patients with HER2-positive breast cancer (ExteNET): a multicentre, randomised, double-blind, placebo-controlled, phase 3 trial. *Lancet Oncol* 2016;17:367-77.

52 Mirza MR, Monk BJ, Herrstedt J, et al. Niraparib maintenance therapy in platinum-sensitive, recurrent ovarian cancer. *N Engl J Med* 2016;375:2154-64.

53 Ledermann J, Harter P, Gourley C, et al. Olaparib maintenance therapy in platinum-sensitive relapsed ovarian cancer. *N Engl J Med* 2012;366:1382-92.

54 Pujade-Lauraine E, Ledermann JA, Selle F, et al. Olaparib tablets as maintenance therapy in patients with platinum-sensitive, relapsed ovarian cancer and a BRCA1/2 mutation (SOLO2/ENGOT-Ov21): a double-blind, randomised, placebo-controlled, phase 3 trial. *Lancet Oncol* 2017;18:1274-84.

55 Robson M, Im SA, Senkus E, et al. Olaparib for metastatic breast cancer in patients with a germline BRCA mutation. *N Engl J Med* 2017;377:523-33.

56 Pilla Reddy V, Bui K, Scarfe G, et al. Physiologically based pharmacokinetic modeling for olaparib dosing recommendations: bridging formulations, drug interactions, and patient populations. *Clin Pharmacol Ther* 2019;105:229-41.

57 Rolfo C, Isambert N, Italiano A, et al. Pharmacokinetics and safety of olaparib in patients with advanced solid tumours and mild or moderate hepatic impairment. *Br J Clin Pharmacol* 2020;86:1807-18.

58 Jänne PA, Yang JC, Kim DW, et al. AZD9291 in EGFR inhibitor-resistant non-small-cell lung cancer. *N Engl J Med* 2015;372:1689-99.

59 Goss G, Tsai CM, Shepherd FA, et al. Osimertinib for pretreated EGFR Thr790Met-positive advanced non-small-cell lung cancer (AURA2): a multicentre, open-label, single-arm, phase 2 study. *Lancet Oncol* 2016;17:1643-52.

60 Mok TS, Wu YL, Ahn MJ, et al. Osimertinib or platinum-pemetrexed in EGFR T790M-positive lung cancer. *N Engl J Med* 2017;376:629-40.

61 Soria JC, Ohe Y, Vansteenkiste J, et al. Osimertinib in untreated EGFR-mutated advanced non-small-cell lung cancer. *N Engl J Med* 2018;378:113-25.

62 Yang JC, Ahn MJ, Kim DW, et al. Osimertinib in pretreated T790M-positive advanced non-small-cell lung cancer: AURA study phase II extension component. *J Clin Oncol* 2017;35:1288-96.

63 Grande E, Harvey RD, You B, et al. Pharmacokinetic study of osimertinib in cancer patients with mild or moderate hepatic impairment. *J Pharmacol Exp Ther* 2019;369:291-99.

64 Finn RS, Martin M, Rugo HS, et al. Palbociclib and letrozole in advanced breast cancer. *N Engl J Med* 2016;375:1925-36.

65 Turner NC, Ro J, André F, et al. Palbociclib in hormone-receptor-positive advanced breast cancer. *N Engl J Med* 2015;373:209-19.

66 San-Miguel JF, Hungria VT, Yoon SS, et al. Panobinostat plus bortezomib and dexamethasone versus placebo plus bortezomib and dexamethasone in patients with relapsed or relapsed and refractory multiple myeloma: a multicentre, randomised, double-blind phase 3 trial. *Lancet Oncol* 2014;15:1195-206.

67 Richardson PG, Schlossman RL, Alsina M, et al. PANORAMA 2: panobinostat in combination with bortezomib and dexamethasone in patients with relapsed and bortezomib-refractory myeloma. *Blood* 2013;122:2331-7.

68 Slingerland M, Hess D, Clive S, et al. A phase I, open-label, multicenter study to evaluate the pharmacokinetics and safety of oral panobinostat in patients with advanced solid tumors and various degrees of hepatic function. *Cancer Chemother Pharmacol* 2014;74:1089-98.

69 Hortobagyi GN, Stemmer SM, Burris HA, et al. Ribociclib as first-line therapy for HR-positive, advanced breast cancer. *N Engl J Med* 2016;375:1738-48.

70 Tripathy D, Im SA, Colleoni M, et al. Ribociclib plus endocrine therapy for premenopausal women with hormone-receptor-positive, advanced breast cancer (MONALEESA-7): a randomised phase 3 trial. *Lancet Oncol* 2018;19:904-15.

71 Slamon DJ, Neven P, Chia S, et al. Phase III randomized study of ribociclib and fulvestrant in hormone receptor-positive, human epidermal growth factor receptor 2-negative advanced breast cancer: MONALEESA-3. *J Clin Oncol* 2018;36:2465-72.

72 Swisher EM, Lin KK, Oza AM, et al. Rucaparib in relapsed, platinum-sensitive high-grade ovarian carcinoma (ARIEL2 Part 1): an international, multicentre, open-label, phase 2 trial. *Lancet Oncol* 2017;18:75-87.

73 Coleman RL, Oza AM, Lorusso D, et al. Rucaparib maintenance treatment for recurrent ovarian carcinoma after response to platinum therapy (ARIEL3): a randomised, double-blind, placebo-controlled, phase 3 trial. *Lancet* 2017;390:1949-61.

74 Kristeleit R, Shapiro GI, Burris HA, et al. A phase I-II study of the oral PARP inhibitor rucaparib in patients with germline BRCA1/2-mutated ovarian carcinoma or other solid tumors. *Clin Cancer Res* 2017;23:4095-106.

75 Migden MR, Guminski A, Gutzmer R, et al. Treatment with two different doses of sonidegib in patients with locally advanced or metastatic basal cell carcinoma (BOLT): a multicentre, randomised, double-blind phase 2 trial. *Lancet Oncol* 2015;16:716-28.

76 Horsmans Y, Zhou J, Liudmila M, et al. Effects of mild to severe hepatic impairment on the pharmacokinetics of sonidegib: a multicenter, open-label, parallel-group study. *Clin Pharmacokinet* 2018;57:345-54.

77 Litton JK, Rugo HS, Ettl J, et al. Talazoparib in patients with advanced breast cancer and a germline BRCA mutation. *N Engl J Med* 2018;379:753-63.

78 Demetri GD, Chawla SP, von Mehren M, et al. Efficacy and safety of trabectedin in patients with advanced or metastatic liposarcoma or leiomyosarcoma after failure of prior anthracyclines and ifosfamide: results of a randomized phase II study of two different schedules. *J Clin Oncol* 2009;27:4188-96.

79 Monk BJ, Herzog TJ, Kaye SB, et al. Trabectedin plus pegylated liposomal doxorubicin in recurrent ovarian cancer. *J Clin Oncol* 2010;28:3107-14.

80 Samuels BL, Chawla S, Patel S, et al. Clinical outcomes and safety with trabectedin therapy in patients with advanced soft tissue sarcomas following failure of prior chemotherapy: results of a worldwide expanded access program study. *Ann Oncol* 2013;24:1703-9.

81 Mayer RJ, Van Cutsem E, Falcone A, et al. Randomized trial of TAS-102 for refractory metastatic colorectal cancer. *N Engl J Med* 2015;372:1909-19.

82 Shitara K, Doi T, Dvorkin M, et al. Trifluridine/tipiracil versus placebo in patients with heavily pretreated metastatic gastric cancer (TAGS): a randomised, double-blind, placebo-controlled, phase 3 trial. *Lancet Oncol* 2018;19:1437-48.

83 Seymour JF, Kipps TJ, Eichhorst B, et al. Venetoclax-rituximab in relapsed or refractory chronic lymphocytic leukemia. *N Engl J Med* 2018;378:1107-20.

84 Roberts AW, Davids MS, Pagel JM, et al. Targeting BCL2 with venetoclax in relapsed chronic lymphocytic leukemia. *N Engl J Med* 2016;374:311-22.

85 Stilgenbauer S, Eichhorst B, Schetelig J, et al. Venetoclax in relapsed or refractory chronic lymphocytic leukaemia with 17p deletion: a multicentre, open-label, phase 2 study. *Lancet Oncol* 2016;17:768-78.

86 Jones JA, Mato AR, Wierda WG, et al. Venetoclax for chronic lymphocytic leukaemia progressing after ibrutinib: an interim analysis of a multicentre, open-label, phase 2 trial. *Lancet Oncol* 2018;19:65-75.

87 DiNardo CD, Pratz K, Pullarkat V, et al. Venetoclax combined with decitabine or azacitidine in treatment-naive, elderly patients with acute myeloid leukemia. *Blood* 2019;133:7-17.

88 Wei AH, Strickland SA, Jr., Hou JZ, et al. Venetoclax combined with low-dose cytarabine for previously untreated patients with acute myeloid leukemia: results from a phase Ib/II study. *J Clin Oncol* 2019;37:1277-84.

89 Edlund H, Lee SK, Andrew MA, et al. Population pharmacokinetics of the BTK inhibitor acalabrutinib and its active metabolite in healthy volunteers and patients with B-cell malignancies. *Clin Pharmacokinet* 2019;58:659-72.

90 James AJ, Smith CC, Litzow M, et al. Pharmacokinetic profile of gilteritinib: a novel FLT-3 tyrosine kinase inhibitor. *Clin Pharmacokinet* 2020;59:1273-90.

91 Masters JC, LaBadie RR, Salageanu J, et al. Pharmacokinetics and safety of glasdegib in participants with moderate/severe hepatic impairment: a phase I, single-dose, matched case-control study. *Clin Pharmacol Drug Dev* 2020:doi: 10.1002/cpdd.897. [online ahead of print].

92 Fan B, Dai D, Cohen M, et al. Effect of mild and moderate hepatic impairment on the pharmacokinetics, safety, and tolerability of a single dose of oral ivosidenib in otherwise healthy participants. *Clin Pharmacol Drug Dev* 2021;10:99-109.

93 Gupta N, Hanley MJ, Venkatakrishnan K, et al. Pharmacokinetics of ixazomib, an oral proteasome inhibitor, in solid tumour patients with moderate or severe hepatic impairment. *Br J Clin Pharmacol* 2016;82:728-38.

94 Calvo E, Azaro A, Rodon J, et al. Hepatic safety analysis of trabectedin: results of a pharmacokinetic study with trabectedin in patients with hepatic impairment and experience from a phase 3 clinical trial. *Invest New Drugs* 2018;36:476-86.
